# Effect of Antioxidant Supplementation on Macular Pigment Optical Density and Visual Functions: A Systematic Review and Network Meta-Analysis of Randomized Controlled Trials

**DOI:** 10.1016/j.advnut.2024.100216

**Published:** 2024-04-04

**Authors:** Weili Hu, Vernice Seah, Vanessa Huang, Jung Eun Kim

**Affiliations:** Department of Food Science and Technology, National University of Singapore, Singapore

**Keywords:** antioxidants, xanthophyll, fatty acid, vitamin, macular pigment optical density, visual acuity, contrast sensitivity, photostress recovery time

## Abstract

Antioxidants are bioactive molecules that function to scavenge free radicals and balance oxidative stress. Although all antioxidants can act as reactive oxygen species scavengers, their efficacy on eye health may vary. Moreover, the comparative effectiveness and potential additive effect between groups of antioxidants, hitherto, have not been systematically studied. A systematic review and network meta-analysis were conducted to investigate the comparative or additive effect of dietary antioxidant supplements on eye health. Four databases (PubMed, Embase, CINAHL, and Cochrane) were searched, and relevant randomized controlled trials were identified. Out of 60 articles selected for systematic review, 38 were included in the network meta-analysis, categorized into 8 distinct antioxidant-supplemented groups and placebo. All groups significantly increased macular pigment optical density and contrast sensitivity at low spatial frequency, whereas only the antioxidant mixture + lutein (L) + fatty acid combination exhibited significant improvements in visual acuity (hazard ratio = –0.15; 95% confidence interval: –0.28, –0.02) and L + zeaxanthin combination for photostress recovery time (hazard ratio = –5.75; 95% confidence interval: –8.80, –1.70). Especially, the L + zeaxanthin + fatty acid combination was ranked best for macular pigment optical density (surface under the cumulative ranking: 99.3%) and second best for contrast sensitivity at low spatial frequency (67.7%). However, these findings should be interpreted with caution due to low quality of evidence, primarily influenced by indirectness and potential publication bias. Overall, antioxidant supplementation was estimated to improve eye health parameters, whereas different combinations of antioxidants may also have varying effects on improving visual health from multiple perspectives.

This study was registered at PROSPERO as CRD42022369250.

**Statement of significance**

This review showed that various combinations of antioxidants would have distinct effects on improving visual health from multiple angles and particularly confirmed the adjunctive role of fatty acids in combination with macular xanthophylls in improving macular pigment optical density.

## Introduction

Vision impairments can have significant and far-reaching consequences for individuals, communities, and society at large. These consequences span from impacting physical and mental well-being to reducing social interactions within the community and decreasing work productivity. A key underlying cause of visual impairment is the accumulation of reactive oxygen species and an increase of oxidative stress status in the eye, resulting in biomolecule damage, including DNA mutation and lipid peroxidation [1]. These cellular damages contribute to the pathogenesis of many eye disorders, such as drusen formation between the retinal pigment epithelium and Bruch’s membrane, leading to age-related macular degeneration (AMD) [2] and degeneration of retinal ganglion leading to glaucoma [3]. Thus, maintaining low oxidative stress status in the eye may be crucial to ensure good eye health and lower risk of eye disorders.

Antioxidants are bioactive molecules that can function to scavenge free radicals and balance oxidative stress. Commonly found natural antioxidants include vitamin C, vitamin E, polyphenols, and carotenoids, as well as enzyme cofactors and minerals such as zinc and selenium [4]. Although all function as reactive oxygen species scavengers, their efficacy on eye health may vary. Previous systematic review and meta-analysis revealed that higher macular xanthophyll [lutein (L), zeaxanthin (Z), and meso-zeaxanthin (MZ)] intake from both supplements and food sources can improve macular pigment optical density (MPOD), and flavonoid supplementation can also improve visual acuity and retinal sensitivity [1,5,6]. However, a recent Cochrane review revealed no association between dietary intake of vitamin C and risk of AMD [7]. Another review by Vishwanathan et al. [8] also reported inconclusive results for zinc supplementation and progression of AMD. Although the consumption of omega (ω)-3 fatty acid (FA) was suggested to have beneficial effects on AMD [9] and dry eyes [10] for its antioxidant behavior to scavenge superoxide [11], clinical trials assessing the potential effects of ω-3 supplementation on reducing risk of developing AMD and alleviating dry eye symptoms were also inconclusive [[12], [13], [14], [15]].

The different efficacies of those antioxidants can be attributed to their physiological functions as well as absorption and bioavailability in relation to biological structures [[16], [17], [18]]. For this reason, researchers suggest that different groups of antioxidants can work cooperatively to enhance eye protective effects [19]. Although supplementation of 1 antioxidant may not show improved eye health, incorporating a mixture of various antioxidant groups resulted in enhanced visual outcomes as the regimen of supplements used in the Age-Related Eye Disease Studies, including vitamin C, vitamin E, beta-carotene, zinc, and ω-3 FA collectively improved visual outcomes and was associated with lowered risk of diseases progression in patients with AMD [20].

Although it has been well established that antioxidants play an important role in regulating oxidative stress status in the eye, the comparative effectiveness and potential additive effect between groups of antioxidants, up to date, have not been systematically studied. Therefore, the objective of this study is to investigate the effect of dietary antioxidant supplements on eye health through a systematic review and network meta-analysis (NMA) of randomized controlled trials.

## Methods

This systematic review and NMA were registered in PROSPERO (www.crd.york. ac.uk/prospero/index.asp, identifier CRD42022369250) and reported in adherence to PRISMA standards for reporting NMAs [21].

### Search strategy

The participant, intervention, comparison, outcome, and study design statement are shown in Table 1. A computerized literature search was independently performed by 2 reviewers (VS and VH) using 4 databases: PubMed, Embase, CINAHL, and Cochrane Library in September 2022, updated in May 2023. Keywords used during the search included vitamins, lipids, FA, minerals, carotenoids, polyphenols, flavonoids, eye diseases, MPOD, glare sensitivity, contrast sensitivity, visual acuity, and oxidative stress. Details of the set of search terms and limiters can be found in Supplemental Table 1.

**TABLE 1:** Description of participant, intervention, comparison, outcome, and study statement

Parameter	Descriptions
Population	Adults (age ≥ 18 y)
Intervention	Groups that consumed antioxidant supplements
Comparison	Groups that received different treatment or placebo
Outcomes	Primary: MPOD Secondary: contrast sensitivity, visual acuity, andphotostress recovery time
Study design	Randomized controlled trials

Abbreviation: MPOD, macular pigment optical density.

### Selection of studies

Independent screening of the title and abstract by both the primary (VS) and secondary (VH) reviewers were conducted based on the exclusion criteria: *1*) nonrandomized controlled trial study design; *2*) study populations with children aged <18 y, pregnant or breastfeeding females, animal or in vitro studies; *3*) primary outcome not reported; and *4*) intervention with nonsupplement sources of antioxidants, i.e., food sources. Any discrepancies during the screening and extraction process were discussed to a consensus between the 2 reviewers and resolved by a third reviewer (WH).

### Data extraction

Two reviewers (VS and VH) independently extracted the following data from each included article: author, publication year; study intervals, number of subjects for each study group, study period, subject age, supplement type, supplement dosage, and pre–post intervention and/or change mean values and SDs for primary outcome MPOD; and secondary outcomes, visual acuity, contrast sensitivity, and photostress recovery time. When additional information was needed, the corresponding authors of the selected articles were contacted.

### Risk of bias and quality of evidence

Risk of bias tool for randomized trials version 2, provided by the Cochrane Handbook for Systematic Reviews of Intervention, was used to evaluate risk of bias in each included article. Low, some concerns or high risks were assigned to the articles in the following domains: randomization process, deviation from intended interventions, missing outcome data, measurement of the outcome, selection of the reported result, and overall bias. Two researchers (WH and VS) independently assessed risk of bias, whereas any discrepancies were resolved with the involvement of the other coauthors. The quality of evidence was assessed using the Grading of Recommendations Assessment, Development, and Evaluation approach, considering study limitations, inconsistency, indirectness, imprecision, and publication bias for all outcomes [22,23].

### Data synthesis and analysis

Direct comparisons between different intervention groups were presented using a network plot for all the reported outcomes [24]. The size of the node is proportional to the number of studies in each specific intervention; the thickness of the line is proportional to the number of comparisons included in the network. Random-effects network meta-analyses were performed, allowing for both direct comparisons—where treatments are compared within the same study—and indirect comparisons—where treatments are compared across different studies, linked by a common comparator. This approach was utilized to determine the pooled relative effect of each intervention group against every other group for each outcome measure of interest [25]. Results were reported as standardized mean differences with a 95% confidence interval (CI) for primary outcome MPOD due to differences in measuring equipment, whereas mean differences were employed for secondary outcomes, visual acuity, contrast sensitivity, and photostress recovery time. The units for secondary outcomes included are as follows: logCS units for contrast sensitivity, time in seconds (s) for photostress recovery time, and logMAR for visual acuity, where conversions of results under Early Treatment for Diabetic Retinopathy Study to logMAR were performed using a previously reported method [26]. Contrast sensitivity data were categorized into low, normal, and high spatial frequencies based on the cycles per degree settings [27]. The assumption of transitivity was evaluated to ensure the validity of comparing treatments through a common comparator, thereby maintaining the coherence of indirect evidence across studies. This evaluation was conducted by comparing the distribution of potential effect modifiers, namely age, BMI (in kg/m^2^), study duration (in wk), percentage of male subjects, and sample size. Sensitivity analysis was conducted by excluding articles ranked as having high overall risk of bias. Global inconsistencies were assessed by comparing the posterior mean deviance of each data point between 2 models: the consistency model assumes that different types of evidence (direct and indirect) agree and can be combined to estimate treatment effects, whereas the inconsistency model checks for disagreement between evidence types, suggesting that treatment effects may differ when directly compared with indirectly [28]. All data analyses were performed under the Bayesian framework and, using the Markov Chain Monte Carlo simulation model as developed in the BUGSnet package (R Stuido, 12.1) [24,29] were employed to produce posterior distributions for each parameter of interest. Vague priors were used based on an assumption of limited or no specific prior knowledge of the visual parameters due to a lack of such information in most articles. Additionally, the heterogeneity found in measurement techniques, population demographics, and experimental conditions across studies is likely to have resulted in a wide range of values. The point estimates were determined as the median values of the posterior distributions resulting from the NMA, and precision measures were derived from the 2.5th and 97.5th percentiles of the posterior distributions. The ranking probabilities of intervention groups were evaluated using the surface under the cumulative ranking (SUCRA) curve. The SUCRA values for a given rank X are interpreted as the probability that a particular treatment is the X-th best among those being compared for improving a specific outcome, as inferred from the posterior distribution [30]. The *P* values were subsequently calculated by examining the proportion of the posterior distribution that was more extreme than the observed effect, taking into account both sides of the distribution if the test was 2-tailed. Statistical significance was noted at *P* value of <0.05. The dmetar package (R Stuido, 12.1) was used to assess direct and indirect evidence as well as publication bias [31].

## Results

### Results of the literature search

**FIGURE 1:**
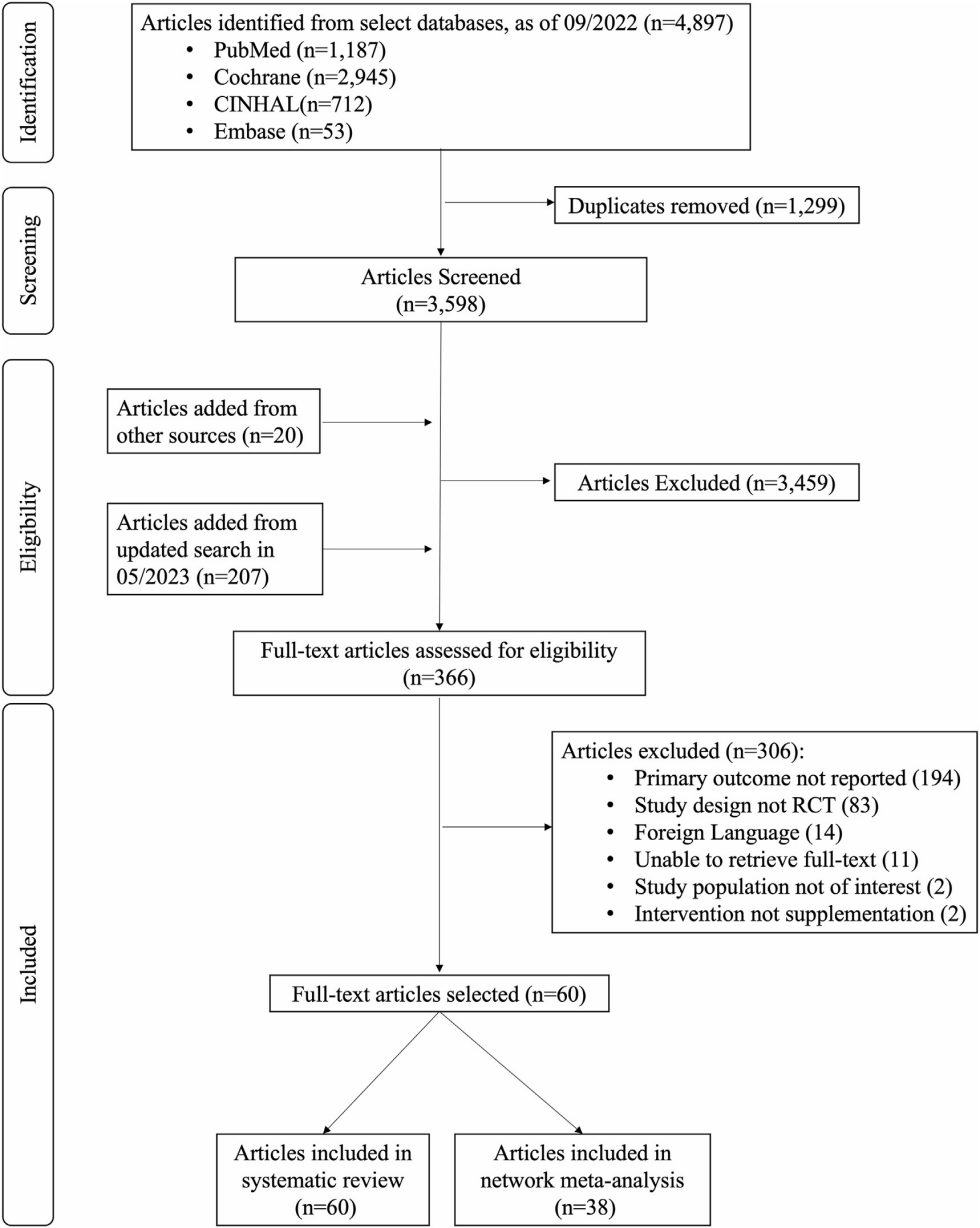
Flow diagram of the literature search process. RCT, randomized controlled trial.

As shown in Figure 1, a total of 4897 articles were initially identified. Of these, 1299 were excluded for duplication. After carefully reviewing the titles and abstracts, another 3257 were excluded, whereas full texts of the 341 remaining articles were further examined to assess their eligibility. An additional 20 articles from other sources were also identified and added. Two hundred seven articles from the updated search in May 2023 were screened. Three hundred six articles were excluded due to following reasons: 194 articles for the primary outcome were not reported, 83 for the study design not being a randomized controlled trial, 14 articles for published language not in English, 11 articles for their full texts could not be retrieved, 2 for the study population not of interest and 2 for study intervention with whole foods instead of supplementation. Of the qualified articles, 60 were used in the systematic review [[32], [33], [34], [35], [36], [37], [38], [39], [40], [41], [42], [43], [44], [45], [46], [47], [48], [49], [50], [51], [52], [53], [54], [55], [56], [57], [58], [59], [60], [61], [62], [63], [64], [65], [66], [67], [68], [69], [70], [71], [72], [73], [74], [75], [76], [77], [78], [79], [80], [81], [82], [83], [84], [85], [86], [87], [88], [89]]. Out of these, only 38 were eligible for the NMA, as some articles were excluded due to inextricable data or the absence of suitable comparison groups [32,35,36,38,40,[44], [45], [46], [47], [48], [49], [50], [51], [52],^55^,^57^,[59], [60], [61], [62], [63], [64], [65], [66],^71^,[73], [74], [75], [76], [77], [78],^80^,^82^,^83^,^85^,^86^,^90^,^91^].

### Study characteristics

The study and subject characteristics of the articles included in this review are presented in Supplemental Table 2. All 60 included articles were of a parallel study design. Among the selected articles, interventions were classified into the following groups: placebo, L, L+Z, L+Z+FA, L+Z+MZ, antioxidant mixture (MIX)+FA+L, MIX+FA+L+Z, MIX+L+Z, and Z, where additional antioxidants or minerals were categorized under MIX. Studies utilizing various supplement types were grouped into combined categories (e.g., L+Z, L+Z+MZ), whereas those using a single supplement type were listed under individual categories. This ensured each study was uniquely classified according to its design. The intervention dosage varied, with L or Z ranging from 2 mg to 20 mg or more. Most FA interventions predominantly incorporated ω-3 FAs, specifically docosahexaenoic acid. However, Bovier et al. [40] was an exception, as the authors did not specify and instead used a mix of n–s FAs. Articles that employed alternative antioxidants outside of the macular xanthophyll or FA groups were categorized with MIX, incorporating supplements such as anthocyanins, minerals, and vitamins. The duration of intervention ranged from 6 to 208 wk, and the mean age of the subjects included in these trials was between 19 and 77 y old. Twenty-three articles included subjects who were past or current smokers, whereas others included healthy or did not mention the smoking status of the subjects. Thirty-three articles reported on healthy subjects, whereas 27 articles recruited subjects with pre-existing eye conditions, with 18 articles conducted for subjects with AMD.

### Network diagrams

**FIGURE 2:**
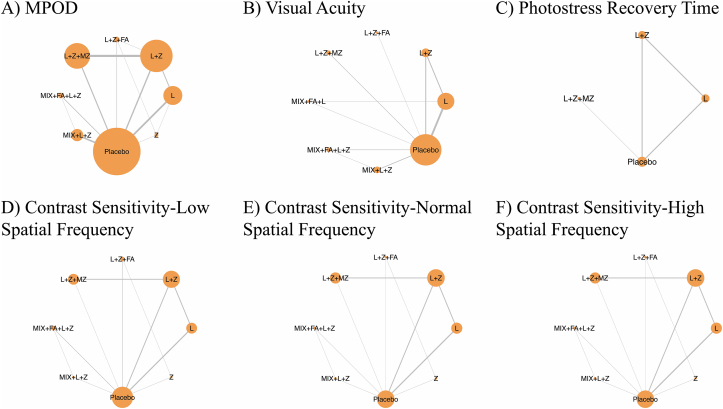
Network diagram for (A) MPOD, (B) visual acuity, (C) photostress recovery time, (D) contrast sensitivity-low spatial frequency, (E) contrast sensitivity-normal spatial frequency, and (F) contrast sensitivity-high spatial frequency. The node size corresponds to the number of studies for each specific intervention, whereas the line thickness represents the number of comparisons within the network. L, lutein; L+Z, lutein + zeaxanthin; L+Z+FA, lutein + zeaxanthin + fatty acid; L+Z+MZ, lutein + zeaxanthin + meso-zeaxanthin; MIX+FA+L, antioxidant mixture + fatty acid + lutein; MIX+FA+L+Z, antioxidant mixture + fatty acid + lutein + zeaxanthin; MIX+L+Z, antioxidant mixture + lutein + zeaxanthin; MPOD, macular pigment optical density; Z, zeaxanthin.

The network diagram of the primary outcome MPOD is shown in Figure 2A. The most common direct comparisons occurred between the L+Z compared with L+Z+MZ intervention, followed by L compared with placebo. Figure 2B–F presents the direct comparisons for network diagrams for the secondary outcomes: visual acuity (Figure 2B), photostress recovery time (Figure 2C), and contrast sensitivity at low, normal, and high spatial frequency (Figure 2D–F, respectively). Details of the number of comparisons between interventions and the number of participants included for each outcome can be found in Supplemental Tables 3–8.

### Outcomes

#### MPOD

**FIGURE 3:**
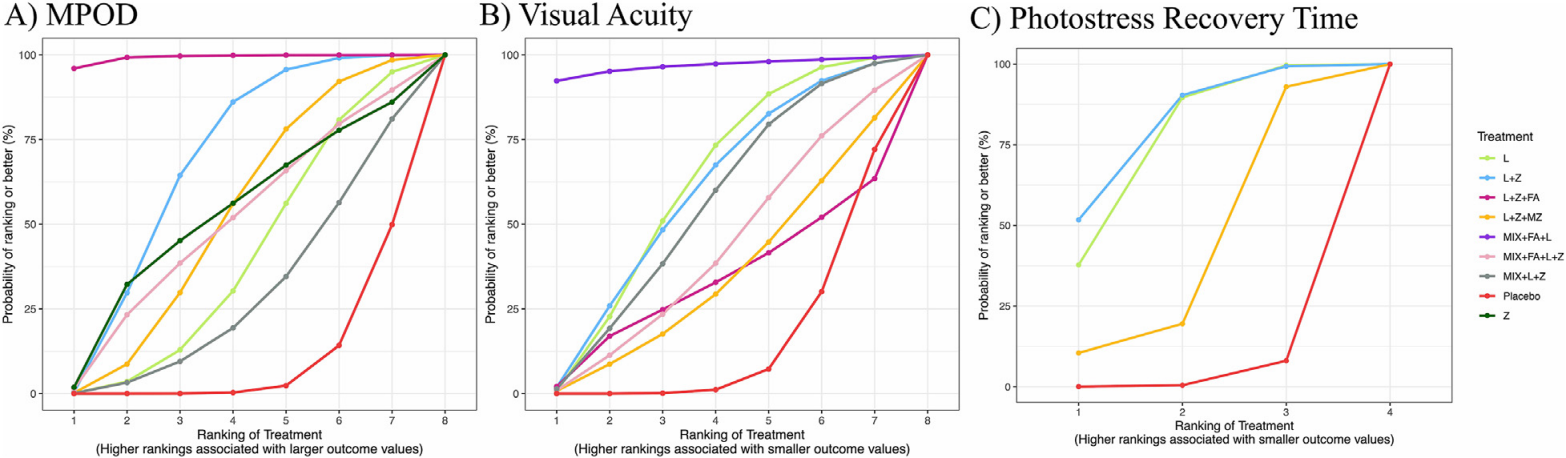
Surface under the cumulative ranking curve plot for (A) MPOD, (B) visual acuity, and (C) photostress recovery time. L, lutein; L+Z, lutein + zeaxanthin; L+Z+FA, lutein + zeaxanthin + fatty acid; L+Z+MZ, lutein + zeaxanthin + meso-zeaxanthin; MIX+FA+L+Z, antioxidant mixture + fatty acid + lutein + zeaxanthin; MIX+L+Z, antioxidant mixture + lutein + zeaxanthin; MPOD, macular pigment optical density; Z, zeaxanthin.

In the NMA, all intervention groups significantly increased MPOD compared with the placebo group. Detailed information about the relative effectiveness of all possible pairs of interventions can be found in the league table (Table 2). Among the different interventions, L+Z+FA had the highest SUCRA (99.3%), followed by L+Z (68.0%) and Z (52.7%), as shown in Figure 3A**.** SUCRA also revealed that the combination of a mixture of antioxidants was ranked lower than that when the respective group was given solely (MIX+L+Z+FA: 50.4% compared with L+Z+FA: 99.3% and MIX+L+Z: 29.1% compared with L+Z: 68.0%). In the comparisons involving FA in combination treatments, notably between L+Z and L+Z+FA as well as between MIX+L+Z and MIX+FA+L+Z, the groups supplemented with FA consistently exhibited a higher ranking.

**TABLE 2:** League table (macular pigment optical density)^1^

Placebo							
–11.50 (–28.24 to 5.20)	L	—	—	—	—	—	—
–22.13 (–37.72 to –6.74)	–10.63 (–30.03 to 8.24)	L+Z	—	—	—	—	—
–62.73 (–93.97 to –31.23)	–51.23 (–85.38 to –16.93)	–40.60 (–73.04 to –8.06)	L+Z+FA	—	—	—	—
–16.54 (–33.96 to 0.63)	–5.05 (–27.12 to 16.60)	15.32 (–9.97 to 40.90)	46.19 (11.60–80.31)	L+Z+MZ	—	—	—
–16.18 (–45.46 to 13.38)	–4.69 (–38.64 to 29.57)	5.94 (–27.15 to 39.00)	46.55 (3.97–89.37)	0.36 (–33.53 to 34.17)	MIX+FA+L+Z	—	—
–6.80 (–26.61 to 12.91)	4.69 (–21.27 to 30.87)	15.32 (–9.97 to 40.90)	55.93 (19.33–92.96)	9.74 (–16.69 to 36.54)	9.38 (–23.74 to 42.68)	MIX+L+Z	—
–18.22 (–55.43 to 18.75)	–6.73 (–44.19 to 30.62)	3.90 (–35.18 to 42.70)	44.51 (3.15–86.58)	–1.68 (–41.94 to 38.18)	–2.04 (–49.83 to 45.07)	–11.42 (–53.76 to 30.85)	Z

Abbreviations: CI, confidence interval, L, lutein; L+Z, lutein + zeaxanthin; L+Z+FA, lutein + zeaxanthin + fatty acid; L+Z+MZ, lutein + zeaxanthin + meso-zeaxanthin; MIX+FA+L+Z, antioxidant mixture + fatty acid + lutein + zeaxanthin; MIX+L+Z, antioxidant mixture + lutein + zeaxanthin; Z, zeaxanthin.

1 The values above the intervention classes correspond to the difference in standardized mean (95% CI) in macular pigment optical density between the row and columns (e.g., the standardized mean difference in macular pigment optical density between placebo and lutein intervention is −11.50).

#### Visual acuity

For visual acuity, only the MIX+FA+L intervention elicited a significant improvement, shown by the decrease in logMAR [hazard ratio (HR) = –0.15; 95% CI: –0.28, –0.02] compared with the placebo, whereas no significant differences were observed between the other groups of interventions (Table 3). The MIX+FA+L (SUCRA: 96.8%) showed the highest probability of being the best treatment in improving visual acuity, followed by L (SUCRA: 61.7%) and L+Z (SUCRA: 59.4%) (Figure 3B).

**TABLE 3:** League table (visual acuity)^1^

Placebo							
0.03 (–0.00 to 0.06)	L	—	—	—	—	—	—
0.03 (–0.01 to 0.07)	–0.00 (–0.04 to 0.04)	L+Z	—	—	—	—	—
0.01 (–0.07 to 0.09)	–0.02 (–0.11 to 0.06)	–0.02 (–0.11 to 0.06)	L+Z+FA	—	—	—	—
0.01 (–0.04 to 0.06)	–0.02 (–0.07 to 0.04)	–0.02 (–0.08 to 0.04)	–0.02 (–0.11 to 0.06)	L+Z+MZ	—	—	—
0.15 (0.02 to 0.28)	0.12 (–0.01 to 0.24)	0.12 (–0.02 to 0.25)	–0.02 (–0.08 to 0.04)	0.13 (–0.01 to 0.27)	MIX+FA+L	—	—
0.02 (–0.03 to 0.06)	–0.01 (–0.07 to 0.04)	–0.01 (–0.07 to 0.05)	0.12 (–0.02 to 0.25)	0.01 (–0.06 to 0.07)	–0.13 (–0.27 to 0.01)	MIX+FA+L+Z	—
0.03 (–0.01 to 0.06)	–0.00 (–0.05 to 0.04)	–0.00 (–0.06 to 0.05)	–0.01 (–0.07 to 0.05)	0.02 (–0.05 to 0.07)	–0.12 (–0.25 to 0.01)	0.01 (–0.04 to 0.06)	MIX+L+Z

Abbreviations: CI, confidence interval; L, lutein; L+Z, lutein + zeaxanthin; L+Z+FA, lutein + zeaxanthin + fatty acid; L+Z+MZ, lutein + zeaxanthin + meso-zeaxanthin; MIX+FA+L, antioxidant mixture + fatty acid + lutein; MIX+FA+L+Z, antioxidant mixture + fatty acid + lutein + zeaxanthin; MIX+L+Z, antioxidant mixture + lutein + zeaxanthin.

1 The values above the intervention classes correspond to the difference in mean (95% CI) in visual acuity between the row and columns (e.g., the mean difference in visual acuity between placebo and lutein intervention is 0.03).

#### Photostress recovery time

Direct comparisons involving L, L+Z, L+Z+MZ, and placebo groups were analyzed for photostress recovery time. Only the L+Z intervention exhibited a significant decrease in photostress recovery time (HR = –5.75; 95% CI: –8.80, –1.70) compared with the placebo, whereas no significant differences were observed between the other groups of interventions (Supplemental Table 9). Between the intervention groups, L+Z had the highest SUCRA (81.2%), followed by L (75.3%) and L+Z+MZ (40.8%) (Figure 3C).

#### Contrast sensitivity

**FIGURE 4:**
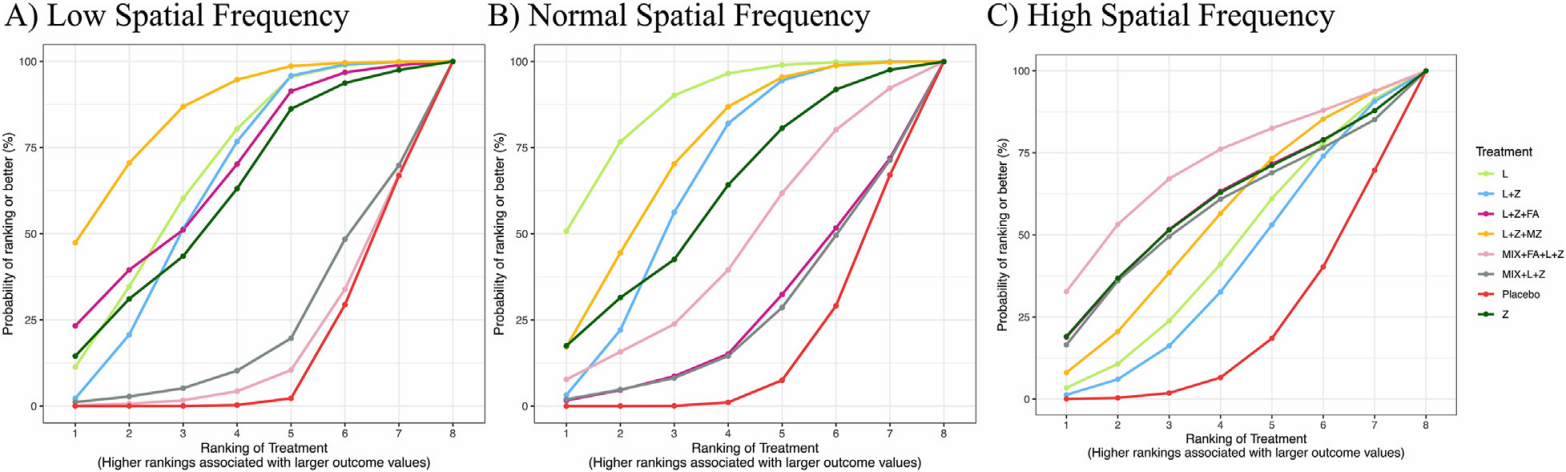
Surface under the cumulative ranking curve plot for contrast sensitivity at (A) low spatial frequency, (B) normal spatial frequency, and (C) high spatial frequency. L, lutein; L+Z, lutein + zeaxanthin; L+Z+FA, lutein + zeaxanthin + fatty acid; L+Z+MZ, lutein + zeaxanthin + meso-zeaxanthin; MIX+FA+L+Z, antioxidant mixture + fatty acid + lutein + zeaxanthin; MIX+L+Z, antioxidant mixture + lutein + zeaxanthin; Z, zeaxanthin.

The ranking probabilities for contrast sensitivity at low, normal, and high spatial frequency were shown in the form of SUCRA plots (Figure 4A–C, respectively) and league tables (Supplemental Tables 10–12). From the league tables, all intervention groups significantly increased contrast sensitivity at low spatial frequency compared with the placebo group; however, such improvements were not observed at normal or high spatial frequency. The L+Z+MZ showed the highest probability to be the best treatment in improving contrast sensitivity at low spatial frequency (SUCRA: 85.4%), followed by the L+Z+FA group (SUCRA: 67.7%), whereas the MIX+ L+Z+FA group (SUCRA: 16.9%) showed to be the least effective compared with the placebo among all interventions.

### Assessment of transitivity

Given the broad range of sample sizes, study durations, and participant gender characteristics, the distributions of potential effect modifiers met the assumption of transitivity. The differences between available direct comparisons were minor for age and BMI. Detailed results of the transitivity analysis can be found in Supplemental Figures 1–6.

### Assessment of inconsistency

Global inconsistency, by plotting of the posterior mean deviance of the individual data points in the inconsistency model against their posterior mean deviance in the consistency model, showed general adherence to the y = x line. This indicates good agreement between the 2 models and suggests that we may proceed with the more parsimonious (consistency) model. The detailed results for each of the reported outcomes can be found in Supplemental Figures 7–12.

### Small study effects, publication bias, and sensitivity analysis

Comparison-adjusted funnel plots were created for all outcomes (Supplemental Figure 13–18). There was no obvious funnel plot asymmetry for MPOD, contrast sensitivity at normal and high spatial frequencies, and photostress recovery time, whereas slightly asymmetric funnel plots were observed with a few outliers for visual acuity and contrast sensitivity at low and high spatial frequencies. Findings from sensitivity analysis were consistent with the results of the main outcome analysis.

### Risk of bias and quality of evidence assessment

The assessments for risk of bias are shown in Supplemental Figure 19. Forty-seven articles were classified as low bias for the randomization process, and 54 trials as low bias for deviations from the intended intervention. Except for Rodriguez et al. [72], assessed as high risk due to a high participant dropout rate, other articles were classified as low risk of missing outcome data. All articles included were assessed as low in the bias for outcome measurement and selection of reported results. Overall, among the 60 articles included in this review, 48 were classified as having overall low risk of bias [33,36,37,[39], [40], [41],[43], [44], [45],[47], [48], [49], [50], [51], [52], [53], [54], [55], [56],[58], [59], [60], [61], [62],^64^,[66], [67], [68], [69], [70], [71],[74], [75], [76], [77], [78], [79], [80], [81], [82], [83], [84], [85],[87], [88], [89], [90], [91]], 11 as some concerns for overall risk of bias [32,34,35,38,42,46,57,63,65,73,86] and 1 article ranked as high risk of bias [72]. Detailed results of the quality of evidence are presented in Supplemental Table 13. Except for contrast sensitivity at normal spatial frequencies, which was rated as having moderate quality of evidence, MPOD, visual acuity, photostress recovery time, and contrast sensitivity at both low and high spatial frequencies were rated as having low quality of evidence. The downgrading was attributed to indirectness due to variations within the population and intervention specifics and to publication bias, as evidenced by funnel plot asymmetry.

## Discussion

Antioxidants, in particular macular xanthophyll, have been well-studied to improve macular pigment accumulation and, subsequently, eye health [5], whereas comparative effectiveness and potential additive effect between groups of antioxidants have not been systematically studied. Findings from this systematic review and NMA of randomized controlled clinical trials suggest that overall supplementation of antioxidants can improve eye MPOD, visual acuity, contrast sensitivity at low spatial frequency, and photostress recovery time compared with not taking antioxidant supplements. Regarding different groups of antioxidant supplementation, L+Z+FA supplementation was found to be the best-ranked intervention for increasing eye MPOD, whereas the MIX+FA+L intervention enhanced visual acuity and L+Z intervention exhibited a significant decrease in photostress recovery time. Moreover, the L+Z+MZ intervention was the best-ranked intervention for contrast sensitivity at low spatial frequency, whereas the L intervention was shown to be the most effective for contrast sensitivity at normal spatial frequency.

In this review, we confirmed the adjunctive role of FAs in combination with macular xanthophyll in improving MPOD. Current evidence indicates that MPOD is a good prognostic marker for disease progression, including AMD, cataract, and macular telangiectasia type 2 [[92], [93], [94]]. MPOD directly reflects macular xanthophyll concentrations, and its bioavailability may increase with FA intake [95], enhancing antioxidant activity, especially in areas susceptible to lipid oxidation [[96], [97], [98]]. Thus, increasing FA consumption may result in a larger ocular domain with a polyunsaturated lipids-embedded membrane, where the xanthophyll co-consumed may selectively accumulate, hence the observed increase in MPOD.

Among xanthophyll-only intervention groups (L, L+Z, L+Z+MZ, Z), we noticed that the L+Z group (SUCRA: 68.0%) is ranked higher than Z (SUCRA: 52.7%), L+Z+MZ (SUCRA: 51.8%), and L (SUCRA: 39.5%) groups. This could be that Z is more concentrated at the retina [99] and exhibits a higher single oxygen quenching rate due to 1 additional conjugated double bond [100], and L has the advantage of a higher lipid membrane solubility, suggesting for higher rate of incorporation into the liposomes and greater bioavailability [101,102]. As for comparisons with the MZ intervention group, mixed results had been reported by published meta-analysis [103,104], and we also failed to observe any significant differences in the HR comparing L+Z+MZ to the other macular xanthophyll intervention groups. As the human body can synthesize MZ from L, more targeted trials comparing between the 2 groups and studies investigating the underlying mechanisms for the retinal interconversion of xanthophylls are needed.

It was also observed that the combination of a mixture of antioxidants with macular xanthophyll was not as effective at enhancing MPOD compared with when the respective group was given solely. One possible explanation could be that studies including vitamins such as vitamin C and vitamin E, minerals such as copper and selenium, as well as other antioxidants such as astaxanthin and anthocyanins were all classified as under the MIX group. Although some of these aforementioned antioxidants can either aid xanthophyll transport and absorption [105] or have already been reported with eye-beneficial effects [20,106], increased dietary intake of others such as minerals (magnesium, zinc) may, in turn, impede xanthophyll bioaccessibility by forming insoluble lipid–soap complex [107]. These structural and chemical differences between the variety of antioxidants may also explain why our findings are inconsistent with a recently published meta-analysis by Wilson et al. [104], which reported no difference in the effect on MPOD between articles of intervention with and without other antioxidants or MZ. However, we must note that there were a larger number of trials conducted with L+Z intervention which may result in the high power.

Regarding visual acuity, unlike our previous MPOD observations, the intervention with a mixture of antioxidants (MIX+FA+L) was shown to be more promising than that in other intervention groups. Although MPOD is directly related to the concentration of xanthophyll concentration at the retina, factor affecting visual acuity also includes illumination, attention, and fatigue [108]. Other nutrients included in the MIX group may affect these other aspects; for example, anthocyanin was reported to improve dark adaptation [109], and magnesium was shown to prevent dry eye disease and decrease eye fatigue [110]. These indirect beneficial impacts on eye health may contribute to the increase in visual acuity and may explain the differences in MPOD results. Photostress recovery time reflects the visual ability of dark adaptation in which the when eyes are bleached by light [111]. In this analysis, the L+Z group had the highest SUCRA between all intervention groups for decreasing photostress recovery time, which aligns with the L+Z group being relatively effective between these groups for increasing MPOD. Enhanced MPOD indicates an increased capacity of the retina to filter short wavelength light and, in turn, leads to improved recovery and shorter photostress recovery time [112].

Contrast sensitivity determines the visibility threshold at a specific spatial frequency, reflecting the ability to detect faintness [113]. In our review, we analyzed contrast sensitivity results in groups of low, normal, and high luminance, where the human visual system is most sensitive to referred to as the normal spatial frequency group (∼6 cpd), whereas requiring higher contrast for lower and higher spatial frequencies [114]. It was observed that antioxidant supplementation could improve contrast sensitivity only in the low-frequency group, whereas such improvement was not significant based on the HR at normal and high spatial frequencies. This increase, in contrast, sensitivity is highly inconsistent with our MPOD observations, where the FA groups were ranked higher than their respective groups. This may be explained by the preferential absorption of macular xanthophyll on blue light reduces the harmful effects of chromatic aberration and enhances contrast sensitivity [115]. Past studies also associated an increase in MPOD with a concomitant increase in contrast sensitivity [40,66]. The lack of conclusive results at high spatial frequencies can be attributed to the rapid sensitivity fall-off for higher spatial frequencies, resulting in difficulties in detection for the observers and a higher requirement to see improved results [116]. We included articles using the Pelli–Robson chart in the normal spatial frequency group; however, the Pelli–Robson chart is not as sensitive in detecting slight improvements due to the large step size in the letters [117] compared with other measuring equipment.

The strength of this study stems from its comprehensive exploration of the potential benefits of various antioxidants, moving beyond the commonly emphasized L and Z. A defining feature of this study is the application of an NMA, an innovative method that integrates both direct and indirect evidence. This approach provides a holistic perspective on the relative efficacy of different interventions, bridging current knowledge gaps. Although the majority of studies have centered on L and Z, a notable disparity was identified in exploring L and Z in combination with other antioxidants. The findings from this study underscore that the integration of FAs can significantly enhance the efficacy of macular xanthophylls in improving eye health, possibly by increasing the bioavailability. Another strength of this study is its focus on supplementation studies, which enhances the control over assessment confounders. Although the form of supplementation—whether esterified or free—can influence nutrient bioavailability, this variability is notably less than that associated with dietary sources. By focusing solely on supplementation, large confounders associated with nutrient bioaccessibility and bioavailability inherent in dietary interventions are effectively minimized. However, some limitations of this review need to be acknowledged. Firstly, populations of different eye statuses were included in the NMA (healthy, AMD, glaucoma, etc.), whereas subjects with pre-existing eye diseases were reported to have poor xanthophyll status and may exhibit differential responses to dietary intake of xanthophyll [5,54]. The researchers were also unable to capture any sources of major heterogeneity and unable to perform further subgrouping on the other potential effect modifiers, such as basal visual acuity and habitual intake of xanthophyll. Significantly, dosage, a key effect modifier, was not accounted for in the current method. This brought forth a notable limitation, where the variance in dosages across treatment groups, though offering a broader view of efficacy, limited direct comparisons between specific dosage amounts. Such discrepancies underscore the need for future dose-specific analyses to determine the optimal dosage range for each treatment group, ensuring more precise clinical recommendations. Another limitation of the study concerns the MIX category’s classification, which, whereas aiming to assess the collective impact of mixed antioxidants on visual health, inadvertently introduced heterogeneity due to the varied antioxidant combinations and dosages. It must also be recognized that certain minerals in these studies might have pro-oxidant effects depending on the context. Future research should aim for more detailed categorization, carefully considering the impacts of individual minerals and their dosages to better understand their distinct roles in eye health. In addition, various measuring techniques for MPOD were included in this review and the use of standardized mean differences rather than weighted mean difference limits the clinical implications for the results.

In conclusion, overall, antioxidant supplementation can improve eye health. Incorporating FAs with macular xanthophyll is more effective at improving eye MPOD, whereas macular xanthophyll combined with antioxidants enhances visual acuity, and the xanthophyll-only group enhances contrast sensitivity mostly. This suggests that different combinations of antioxidants work differently in improving visual health in multiple perspectives.

## Author contributions

The authors’ responsibilities were as follows – JEK, WH: conceived and designed the study; WH, VS, VH: screened, collected, and analyzed the data; WH: wrote the manuscript; JEK: supervised and revised the manuscript; JEK, WH: had primary responsibility for the final content; and all authors: read and approved the final manuscript.

## Conflict of interest

The authors report no conflicts of interest.

## Funding

This research was funded by the National University of Singapore.

## Data availability

Data described in the manuscript, code book, and analytic code will be made available upon request.
