# Supplementary material for: Effect of Antioxidant Supplementation on Macular Pigment Optical Density and Visual Functions: A Systematic Review and Network Meta-Analysis of Randomized Controlled Trials
Source: Adv Nutr. 2024 Apr 4;15(5):100216. doi: 10.1016/j.advnut.2024.100216 (PMC11052915; doi:10.1016/j.advnut.2024.100216)
Supplement: Multimedia component 1 [file mmc1.docx]

**On-line Supplemental Material**

“Effect of Antioxidant Supplementation on Macular Pigment Optical Density and Visual Functions: A Systematic Review and Network Meta-Analysis of Randomized Controlled Trials” by Weili Hu *et al*.

**Supplemental Table 1.** Search Strings and Limits for Each Database

| **Database** | **Search String & Limits** |
| --- | --- |
| PubMed | (“Vitamins”[MeSH] OR vitamins[Title/Abstract] OR Vitamin A[Title/Abstract] OR Vitamin C[Title/Abstract] OR Vitamin E[Title/Abstract] OR “Lipids”[MeSH] OR “Fatty acids”[MeSH] OR Fatty acids[Title/Abstract] OR DHA[Title/Abstract] OR ALA[Title/Abstract] OR EPA[Title/Abstract] OR “Minerals”[MeSH] OR Minerals[Title/Abstract] OR Zinc[Title/Abstract] OR Zinc oxide[Title/Abstract] OR Selenium[Title/Abstract] OR Copper[Title/Abstract] OR “Carotenoids”[MeSH] OR Carotenoids[Title/Abstract] OR Lutein[Title/Abstract] OR Lycopene[Title/Abstract] OR Zeaxanthin[Title/Abstract] OR Beta carotene[Title/Abstract] OR meso-zeaxanthin[Title/Abstract] OR “Polyphenols”[MeSH] OR Polyphenols[Title/Abstract] OR “Flavonoids”[MeSH] OR Flavonoids[Title/Abstract] OR Anthocyanins[Title/Abstract] OR Catechin[Title/Abstract]) AND (“Eye diseases”[MeSH] OR eye diseases[Title/Abstract] OR Macular degeneration[Title/Abstract] OR Cataract[Title/Abstract] OR Retinopathy[Title/Abstract] OR Refractive errors[Title/Abstract] OR Glaucoma[Title/Abstract] OR “Contrast sensitivity”[MeSH] OR Contrast sensitivity[Title/Abstract] OR Macular pigment optical density[Title/Abstract] OR “Visual acuity”[MeSH] OR Visual acuity[Title/Abstract] OR Glare sensitivity[Title/Abstract] OR Photo stress recovery time[Title/Abstract]) *Filters applied: Randomized Controlled Trial, English,Human* |
| Embase | (vitamins:ti,ab,kw OR 'vitamin a':ti,ab,kw OR 'vitamin c':ti,ab,kw OR 'vitamin e':ti,ab,kw OR 'lipid':ti,ab,kw OR 'fatty acid' OR 'dha' OR 'icosapentaenoic acid' OR 'linolenic acid' OR 'mineral' OR 'zinc' OR 'selenium' OR 'copper' OR 'carotenoid' OR 'xanthophyll' OR 'beta carotene' OR 'lycopene' OR 'zeaxanthin' OR 'meso zeaxanthin' OR 'polyphenol' OR 'flavonoid' OR 'anthocyanin' OR 'catechin') AND ('eye disease' OR 'macular degeneration' OR 'cataract' OR 'retinopathy' OR 'refraction error' OR 'glaucoma' OR 'contrast sensitivity' OR 'visual pigment' OR 'optical density' OR 'visual acuity’) AND [embase]/lim NOT ([embase]/lim AND [medline]/lim) AND 'randomised controlled trial'/de AND ([adult]/lim OR [aged]/lim OR [middle aged]/lim OR [very elderly]/lim OR [young adult]/lim) AND 'article'/it AND [english]/lim AND [humans]/lim |
| Cochrane | ((MesH “Contrast Sensitivity”) OR (contrast sensitivity):ti,ab,kw) OR (glare sensitivity):ti,ab,kw) OR (MesH “Visual Acuity”) OR (macular pigment optical density):ti,ab,kw) OR (macular degeneration):ti,ab,kw) OR (visual acuities):ti,ab,kw) OR (cataracts):ti,ab,kw) OR (glaucoma):ti,ab,kw) OR (photostress recovery):ti,ab,kw) OR (refractive errors):ti,ab,kw) OR (MesH “Eye Diseases”))AND ((MesH “Lipids”) OR (DHA,ALA,EPA):ti,ab,kw) OR (MesH “Vitamins”) OR (vitamin a OR vitamin c OR vitamin e):ti,ab,kw) OR (zinc OR copper OR selenium):ti,ab,kw) OR (MesH “Minerals”) OR (MesH “Carotenoids”) OR (lutein ORbeta carotene OR lycopene OR zeaxanthin' OR meso zeaxanthin):ti,ab,kw) OR (MesH “Flavonoids”) OR (MesH “Polyphenols”) OR (anthocyanins):ti,ab,kw) in Trials |
| CINAHL | ((MH Vitamins) OR (AB ("vitamin a") OR ("vitamin c") or ("vitamin e"))) OR (MH( Lipids or "Fatty acids")) OR (AB (DHA) or (EPA) or (ALA)) OR (MH minerals) OR (AB (zinc) OR (selenium) OR (Copper)) OR (MH (carotenoids) OR (AB (Lutein) OR (Lycopene) OR (Zeaxanthin) OR ("Beta carotene") OR ("meso-zeaxanthin")) OR (MH (Polyphenols) OR (Anthocyanins) OR (Flavonoids))) AND ((MH "eye disease") OR (AB ("eye diseases") OR ("Macular degeneration") OR ("Cataract") OR ("Retinopathy") OR ("Refractive errors") OR ("Glaucoma") OR (“Contrast sensitivity”) OR "(Contrast sensitivity") OR ("Macular pigment optical density") OR (“Visual acuity”) OR ("Glare sensitivity") OR ("Photo stress recovery time")))**Limiters** - English Language, Randomized Controlled Trials, Humans, Adults; Exclude MEDLINE |

**Supplemental Table 2.** Baseline characteristics of the included RCTs

| ID | Author (Year) | Group | Intervention | Study Duration (weeks) | Sample Size (n) | Mean Age (y) | Mean BMI (kg/m^2^) | %Male | Smokers | Eye  Condition | Reported Outcomes |
| --- | --- | --- | --- | --- | --- | --- | --- | --- | --- | --- | --- |
|  |  |  |  |  |  |  |  |  |  |  |  |
|  |  |  |  |  |  |  |  |  |  |  |  |
| 1 | Akuffo (2015) | Group 1 | L: 20 mg Z: 0.86 mg | 156 | 17 | 65 | 25.5 | 29.4 | Yes (Not significant among groups) | Early AMD | MPOD, CS, VA |
|  |  | Group 2 | MZ: 10 mg  L: 10 mg  Z: 2 mg | 156 | 21 | 64 | 27.1 | 38.1 |  |  |  |
|  |  | Group 3 | MZ: 17 mg  L: 3 mg Z: 2 mg | 156 | 14 | 70 | 25.2 | 35.7 |  |  |  |
| 2 | Arnold (2013) | Placebo | - | 52 | 40 | 68 | 27.1 | 47.5 | - | Non-exudative forms of AMD | MPOD, Serum lutein, zeaxanthin and meso-zeaxanthin |
|  |  | Group 1 | L: 10mg Z: 1mg DHA: 100 mg EPA: 30mg | 52 | 50 | 69 | 27.9 | 42.0 |  |  |  |
|  |  | Group 2 | L: 20 mg Z: 2 mg DHA: 200 mg EPA: 60mg | 52 | 55 | 70 | 28.2 | 48.1 |  |  |  |
| 3 | Azar (2017) | Group A Placebo | - | 32 | 23 | 72.3 | 25.7 | 47.8 | Yes (Not significant among groups) | Without any retinal pathology who underwent cataract surgery 1 month previously | MPOD |
|  |  | Group A Treatment | L: 5 mg  Z: 1 mg | 32 | 24 | 73.04 | 25.2 | 33.3 |  |  |  |
|  |  | Group B Placebo | - | 32 | 39 | 77.26 | 26.2 | 43.6 |  |  |  |
|  |  | Group B Treatment | L: 5 mg  Z: 1 mg | 32 | 40 | 76.35 | 25.6 | 40.0 |  |  |  |
| 4 | Bernstein (2004) | Placebo | - | 17 | 10 | Below 61 | - | - | Did not mention | Preserved central fixation (typically a visual acuity of 20/80 or better) | MPOD and Serum lutein |
|  |  | Lutein | L: 20mg | 17 | 8 | Below 61 | - | - |  |  |  |
| 5 | Berrow (2016) | Untreated | - | 40 | 27 | 43.93 | - | - | Yes (Not significant among groups) | Healthy | MPOD, CS, VA. |
|  |  | Treated | Vit C: 150mg Copper: 400ug Vit E -15mg Zinc -20mg L: 12mg Z: 0.6mg Omega-3 - 1080mg | 40 | 25 | 48.68 | - | - |  |  |  |
| 6 | Berson (2010) | Control | Control | 208 | Control: 88 | Control: 38 | - | Control: 45.22 | - | Retinitis Pigmentosa | MPOD, Serum lutein |
|  |  | Lutein 3.3mg | Lutein 3.3mg + 15 000 IU vit A (retinyl palmitate ) |  | Lutein group: 75 | Lutein group: 48 | - | Lutein group: 52.73 |  |  |  |
|  |  | Lutein 6.6mg | Lutein 6.6mg + 15 000 IU vit A (retinyl palmitate ) |  |  |  | - |  |  |  |  |
|  |  | Lutein 13mg | Lutein 13mg + 15 000 IU vit A (retinyl palmitate ) |  |  |  | - |  |  |  |  |
| 7 | Bone (2007) | Control | Small amount of vegetable oil | 17 | 9 | 22.1 | - | 55.6 | No | Healthy | MPOD, Serum lutein, zeaxanthin and meso-zeaxanthin |
|  |  | Treatment | MZ: 14.9 mg L: 5.5mg Z: 1.4mg | 17 | 10 | 30.5 | - | 80.0 |  |  |  |
| 8 | Bone (2010) | Placebo | - | 20 | 10 | - | - | 60.0 | No | Healthy | MPOD, Serum lutein |
|  |  | Lutein 5mg | L: 5mg | 20 | 17 | - | - | 47.1 |  |  |  |
|  |  | Lutein 10mg | L: 10mg | 20 | 22 | - | - | 68.2 |  |  |  |
|  |  | Lutein 20mg | L: 20mg | 20 | 24 | - | - | 58.3 |  |  |  |
|  |  | Lutein 20mg >50yo | L: 20mg >50yo | 20 | 14 | - | - | 35.7 |  |  |  |
| 9 | Bovier (2015) | Placebo | - | 17 | 15 | 18-32 | - | - | - | Healthy | MPOD, CS |
|  |  | Zeaxanthin | Z: 20mg/day | 17 | 29 | 18-32 | - | - |  |  |  |
|  |  | Multi | Z: 6mg/day L: 8mg/day Mixed n-s fatty acids: 190mg/day | 17 | 25 | 18-32 | - | - |  |  |  |
| 10 | Ceravolo (2019) | Placebo |  | 52 | 15 | 70.87 | 27.7 | 26.7 | - | - | MPOD |
|  |  | L+Z | L: 10 mg Z: 2mg | 52 | 35 | 72.51 | 26 | 48.6 |  |  |  |
| 11 | Choi (2017) | 10mg Z | Z: 10mg/day | 104 | 3 | 55 | - | 0.0 | - | Macular telangiectasia type 2 (MacTel) | VA, CS, MPOD, Serum zeaxanthin |
|  |  | 20mg Z | Z: 20mg/day | 104 | 5 | 61.6 | - | 20.0 |  |  |  |
| 12 | Connolly (2011) | Placebo | - | 26 | 17 | 45 | 26.8 | 40.9 | Yes (Not significant among groups) | Healthy | MPOD, Serum lutein and zeaxanthin |
|  |  | Intervention | MZ: 10.6 mg L: 5.9 mg  Z: 1.2 mg | 26 | 18 | 43 | 27.2 | 36.4 | - |  |  |
| 13 | Davey (2020) | Control | - | 26 | 15 | 67.9 | - | ALL: 37.5 | - | Ocular normal individuals. | MPOD, VA, CS |
|  |  | LM-group | Mixture of antioxidant^1^  Lutein: 15mg Zeaxanthin: 3mg Meso-zeaxanthin: 10mg Astaxanthin: 4000 mcg Fish oil concentrate: 1260mg DHA: 675mg EPA: 230mg | 26 | 25 | 69.9 | - |  |  | Fine retinal drusen sub-clinical AMD |  |
|  |  | PV-group | L: 10mg Z: 2mg Vit C: 500mg Vit E: 400 IU Zinc: 80mg Copper: 2mg | 26 | 16 | 68.4 | - |  |  |  |  |
| 14 | Dawczynski (2013) | Placebo | - | 52 | 40 | ALL: 69 | ALL: 27.8 | ALL: 45.5 | Yes 27.6% | Non-exudative AMD | MPOD, VA |
|  |  | D1 | L; 10mg Z: 1mg Fish oil: 255mg DHA: 100mg EPA: 30mg Antioxidants: 60 mg vitamin C, 20 mg vitamin E, 10 mg zinc, 0.25 mg copper | 52 | 50 |  |  |  |  |  |  |
|  |  | D2 | L: 20mg Z: 2mg Fish oil: 500 mg DHA: 200mg EPA: 60mg Antioxidant: 120 mg vitamin C, 40 mg vitamin E, 20 mg zinc, 0,5 mg copper | 52 | 55 |  |  |  |  |  |  |
| 15 | Fernyhough Culver, Melinda (2018) | Placebo | L: 2mg | 26 | 13 | 19.23 | - | 38.46 | No | Healthy | MPOD, Serum lutein and zeaxanthin |
|  |  | Lutein/Zeaxanthinc Isomers | - | 26 | 35 | 19.26 | - | 45.71 |  |  |  |
| 16 | Forte (2017) | Group 1 | epilutein: 8mg  L: 2mg | 8 | 21 | 69.4 | - | 462.7 | Yes (Not significant between the groups) | Early-stage AMD | MPOD, VA |
|  |  | Group 2 | L:10mg | 8 | 16 | 72 | - | 480.0 |  |  |  |
| 17 | García (2013) | Placebo | - | 52 | 21 | 67.8 | 24.8 | 423.8 | - | Early-stage AMD | MPOD, CS, VA |
|  |  | Treatment | L:12mg Z: 0.6mg DHA: 280mg | 52 | 23 | 69.2 | 25.2 | 432.5 |  |  |  |
| 18 | Hammond, B.R.; Stephen Miller, L. (2017) | Placebo | - | 52 | 15 | 70.93 | - | 26.7 | - | - | MPOD, Serum lutein and zeaxanthin |
|  |  | Treatment | L: 10 mg Z: 2mg | 52 | 36 | 72.51 | - | 47.2 |  |  |  |
| 19 | Hammond, BR; Fletcher, LM (2014) | Placebo | - | 57 | 56 | 22.7 | 22.8 | 39.3 | No | Healthy | MPOD, Photostress recovery time |
|  |  | Treatment | L: 10mg Z: 2mg for 6 months | 57 | 53 | 23.7 | 22.9 | 41.5 |  |  |  |
| 20 | Huang (2013) | Placebo | - | 48 | 27 | 69 | 25.24 | 40.7 | Yes (Not significant among groups) | Early AMD | MPOD, Serum lutein and zeaxanthin |
|  |  | LL | L: 10mg | 48 | 26 | 69.9 | 24.32 | 38.5 |  |  |  |
|  |  | HL | L: 20mg | 48 | 27 | 69 | 25.14 | 44.4 |  |  |  |
|  |  | LZ | L: 10mg  Z: 10mg | 48 | 27 | 68.6 | 24.49 | 44.4 |  |  |  |
| 21 | Huang-BJO (2015) | Placebo | - | 104 | 28 | 69 | 24.8 | 39.3 | Yes (Not significant among groups) | Early AMD | MPOD, CS, VA, Photostress recovery time |
|  |  | 10mg Lutein | L: 10mg | 104 | 26 | 69.7 | 24.1 | 34.6 |  |  |  |
|  |  | 20mg Lutein | L: 20mg | 104 | 27 | 69.3 | 25.1 | 51.9 |  |  |  |
|  |  | 10mg Lutein and 10 mg Zeaxanthin | L: 10mg  Z: 10mg | 104 | 27 | 68.5 | 24.6 | 44.4 |  |  |  |
| 22 | Huang-BMRI (2015) | Placebo | - | 104 | 28 | 69 | 24.8 | 39.3 | Yes (Not significant among groups) | Early AMD | MPOD |
|  |  | 10mg Lutein | L: 10mg | 104 | 26 | 69.7 | 24.1 | 34.6 |  |  |  |
|  |  | 20mg Lutein | L: 20mg | 104 | 27 | 69.3 | 25.1 | 51.9 |  |  |  |
|  |  | 10mg Lutein and 10 mg Zeaxanthin | L: 10mg  Z: 10mg | 104 | 27 | 68.5 | 24.6 | 44.4 |  |  |  |
| 23 | Johnson (2008) | Placebo | - | 17 | 10 | 68 | 23.8 | 0 | No | Healthy | MPOD, Serum lutein and zeaxanthin |
|  |  | DHA | DHA: 800 mg | 17 | 14 | 68 | 24.5 | 0 |  |  |  |
|  |  | Lutein | L: 12mg | 17 | 11 | 65 | 24.6 | 0 |  |  |  |
|  |  | Lutein + DHA | L: 12mg DHA: 800mg | 17 | 14 | 68 | 27 | 0 |  |  |  |
| 24 | Kan (2020) | Placebo | - | 13 | 76 | 37.4 | 24.1 | 17.1 | - | Emmetropiaor low-to-middle myopia | MPOD |
|  |  | 6mg | LE : 12mg Z: 1.2 mg Chrysanthemum extract: 75mg Goji berry extract: 75mg Blackcurrent extract: 100mg | 13 | 76 | 39.1 | 24.4 | 18.4 |  |  |  |
|  |  | 10mg | LE: 20mg Z: 2mg Chrysanthemum extract: 125mg Goji berry extract: 125mg Blackcurrent extract: 167mg | 13 | 75 | 37.8 | 24.1 | 30.7 |  |  |  |
|  |  | 14mg | LE: 28mg Z: 2.8mg Chrysanthemum extract: 175mg Goji berry extract: 175mg Blackcurrent extract: 233mg | 13 | 76 | 38.7 | 23.5 | 22.4 |  |  |  |
| 25 | Kizawa (2021) | Placebo | - | 6 | 20 | 36.1 | 22.1 | - | - | Eye fatigue | MPOD, VA |
|  |  | Active | Anthocyanin: 36mg Astaxanthin: 3mg Lutein: 5mg | 6 | 20 | 37.1 | 22.3 | - | - |  |  |
| 26 | Korobelnik (2017) | Placebo | - | 52 | 60 | 55.8 | 24.8 | 35.0 | Yes (Not significant among groups) | First-generation offspring of parents with neovascular AMD | MPOD, Plasma lutein and zeaxanthin |
|  |  | L+Z | L: 10 mg Z: 2 mg Vit C: 180 mg Vit E: 30 mg zinc: 15 mg Copper: <1.0 mg Resveratrol:1.0 mg Fish oil: 0.66 mg that included 50% ω-3 | 52 | 60 | 57.6 | 24.8 | 21.7 | - |  |  |
| 27 | Kvansakul (2006) | Control |  | 52 | 18 | - | - | - | - | Healthy | MPOD |
|  |  | L | L:10mg | 52 | 16 | - | - | - |  |  |  |
|  |  | Z | Z: 10mg | 52 | 19 | - | - | - |  |  |  |
|  |  | L+Z | L:10mg Z:10mg | 52 | 20 | - | - | - |  |  |  |
| 28 | Landrum (2012) | Placebo | - | 24 | 10 | - | - | - | - | Healthy | MPOD, Serum lutein |
|  |  | Lutein Diacetate | Free lutein: 20mg | 24 | 10 | - | - | - |  |  |  |
|  |  | Lutein | Free lutein: 20mg | 24 | 10 | - | - | - |  |  |  |
| 29 | Loughman (2012) | Placebo | - | 26 | 12 | 46 | 26 | 52.8 | - | Healthy | MPOD, Serum lutein, zeaxanthin, and meso-zeaxanthin, CS |
|  |  | 1 | L: 20mg Z: 2mg | 26 | 12 | 56 | 27 |  |  |  |  |
|  |  | 2 | L: 10mg Z: 2mg MZ: 10mg | 26 | 12 | 51 | 25 |  |  |  |  |
| 30 | Loughman (2021) | Placebo | - | 78 | 20 | 62.89 | 26.6 | 50 | Yes (Not significant among groups) | Open Angle Glaucoma | MPOD, VA |
|  |  | Treatment | L: 10 mg  Z: 2 mg  MZ: 10 mg | 78 | 42 | 66.23 | 28 | 66.7 |  |  |  |
| 31 | Ma, L (2012) | Placebo | - | 48 | 27 | 68.9 | 25.2 | 40.7 | Yes (Not significant among groups) | Probable AMD | MPOD, VA, CS, Photostress recovery time |
|  |  | 10mg Lutein | L: 10mg | 48 | 26 | 69.9 | 24.3 | 38.5 |  |  |  |
|  |  | 20mg Lutein | L: 20mg | 48 | 27 | 69 | 25.1 | 44.4 |  |  |  |
|  |  | Lutein + Zeaxanthin | L: 10mg Z: 10mg | 48 | 27 | 68.6 | 24.5 | 44.4 |  |  |  |
| 32 | Machida (2020) | Placebo | vegetable oil: 180mg | 16 | 31 | 41.1 | 21.3 | 32.3 | No | Healthy | MPOD, Serum lutein, CS |
|  |  | Lutein | Marigold dye preparation (lutein-free form): 30mg(6mg) | 16 | 27 | 42.61 | 20.9 | 33.3 |  |  |  |
| 33 | Murray (2013) | Placebo | - | 52 | 37 | 69.1 | - | 33.3 | Yes (Not significant among groups) | Early AMD | MPOD, VA |
|  |  | Lutein | Lutein ester: 10 mg | 52 | 36 | 71.9 | - | 44.4 |  |  |  |
| 34 | Nolan (2012) | Group 1 | L: 20mg Z: 2mg | 8 | 10 | 51 | - | 30.0 | Yes (Not significant among groups) | Healthy | MPOD |
|  |  | Group 2 | MZ: 10mg L: 10mg Z: 2mg | 8 | 10 | 56 | - | 30.0 |  |  |  |
|  |  | Group 3 | MZ: 17mg L: 3mg Z: 2mg | 8 | 10 | 35 | - | 30.0 |  |  |  |
| 35 | Nolan (2016) | Placebo | - | 52 | 47 | 46.49 | 26.3 | 53.2 | Yes (Not significant among groups) | Healthy | MPOD, Serum lutein, zeaxanthin and meso-zeaxanthin CS, VA |
|  |  | Intervention | L: 10mg Z: 2mg MZ: 10mg | 52 | 48 | 44.83 | 27.3 | 47.9 |  |  |  |
| 36 | Obana (2015) | FloraGLO Group | L: 10.5mg Z: 0.96mg | 26 | 18 | 40.7 | 23 | 50.0 | Yes (Not significant among groups) | Healthy | MPOD, Serum lutein |
|  |  | XanMax Group | L: 10.4mg Z: 1.25mg | 26 | 18 | 42.2 | 22.1 | 50.0 |  |  |  |
| 37 | Richer SP (2011) | Faux Placebo | L: 9mg | 52 | 9 | 73.9 | 29.1 | ALL: 95 | Yes (Not significant among groups) | Atrophic AMD | MPOD, VA |
|  |  | Zx | Z: 8mg | 52 | 21 | 74.4 | 29.8 | ALL: 96 |  |  |  |
|  |  | Zx+L | Z: 8mg L: 9mg | 52 | 21 | 75.8 | 28.6 | ALL: 97 |  |  |  |
| 38 | Richer, S; Devenport, J (2007) | Placebo | - | 52 | ALL: 76 | ALL: 74.7 | - | ALL: 4.44 | - | Atrophic AMD | MPOD |
|  |  | Lutein | L: 10mg | 52 | ALL: 77 | ALL: 74.8 | - | ALL: 4.45 |  |  |  |
|  |  | Lutein + Antioxidants | L: 10mg broad spectrum of antioxidants in a preparation including vitamins, minerals, amino acids, andbioflavonoid | 52 | ALL: 78 | ALL: 74.9 | - | ALL: 4.46 |  |  |  |
| 39 | Richer, Stuart (2004) | Placebo | - | 56 | 31 | 76.1 | 27.3 | 0.7 | Yes (Not significant among groups) | Atrophic AMD | MPOD, CS, VA |
|  |  | Group 1 | L: 10mg | 56 | 29 | 74.4 | 28.5 | 0.7 |  |  |  |
|  |  | Group 2 | L: 10mg antioxidants, vitamins minerals | 56 | 30 | 73.5 | 30.4 | 0.7 |  |  |  |
| 40 | Richer, Stuart (2021) | Placebo | - | 26 | 9 | 65.7 | 26.3 | 77.8 | Yes (Not significant among groups) | Healthy | MPOD, Photostress recovery time |
|  |  | Active | Z: 14mg L: 7mg antioxidants, vitamins minerals, fish oil | 26 | 24 | 61.6 | 31.3 | 79.2 |  |  |  |
| 41 | Rodriguez (2006) | Placebo | - | 26 | 6 | ALL: 22–39 | - | 100 | - | Healthy | MPOD |
|  |  | PC | L: 10mg Z: 10mg | 26 | 5 | ALL: 22–39 | - | 100 |  |  |  |
|  |  | L | L: 10mg | 26 | 3 | ALL: 22–39 | - | 100 |  |  |  |
|  |  | LL | L: 20mg | 26 | 3 | ALL: 22–39 | - | 100 |  |  |  |
|  |  | Z | Z: 10mg | 26 | 5 | ALL: 22–39 | - | 100 |  |  |  |
|  |  | ZZ | Z: 20mg | 26 | 5 | ALL: 22–39 | - | 100 |  |  |  |
|  |  | C | L: 10mg Z: 10mg | 26 | 5 | ALL: 22–39 | - | 100 |  |  |  |
| 42 | Sabour-Pickett (2014) | Group 1 | L: 20 mg Z: 2 mg | 52 | 17 | 65 | 25.5 | 5 | Yes (Not significant among groups) | Early AMD | MPOD, CS |
|  |  | Group 2 | L: 10mg Z: 2mg MZ: 10mg | 52 | 21 | 64 | 27.1 | 8 |  |  |  |
|  |  | Group 3 | L: 3mg Z: 2mg MZ: 17mg | 52 | 14 | 70 | 25.2 | 5 |  |  |  |
| 43 | Sawa (2014) | Placebo | - | 17 | 19 | 46.6 | - | 16 | - | Central Serous Chorioretinopathy | MPOD, Plasma lutein |
|  |  | Lutein | L: 20mg | 17 | 20 | 51.2 | - | 19 |  |  |  |
| 44 | Sawa (2020) | Beeswax | L: 20mg lutein Z: 3mg | 26 | 20 | 69.5 | 22.8 | 13 | Yes (Not significant among groups) | Unilateral exudative AMD | MPOD, Plasma lutein, CS |
|  |  | Glycerol fatty acid esters | L: 20mg lutein Z: 3mg | 26 | 19 | 72 | 23.8 | 11 |  |  |  |
| 45 | Schalch (2007) | P | - | 0-26 | 23 | 24.39 | 23.4 | 23 | - | Healthy | MPOD, Plasma lutein and zeaxanthin |
|  |  | L | L: 10mg | 0-26 | 23 | 26.96 | 24.4 | 23 |  |  |  |
|  |  | Z | Z: 10mg | 0-26 | 23 | 26.26 | 24.1 | 23 |  |  |  |
|  |  | C (L+Z) | L: 10mg Z: 10mg | 0-26 | 23 | 26.13 | 24.2 | 23 |  |  |  |
|  |  | PP | - | 30-56 | - | - | - | - |  |  |  |
|  |  | LL | L: 20 mg | 30-56 | - | - | - | - |  |  |  |
|  |  | ZZ | Z: 20mg | 30-56 | - | - | - | - |  |  |  |
|  |  | CC | L: 20mg Z: 20mg | 30-56 | - | - | - | - |  |  |  |
| 46 | Stringham, JM-EER (2016) | Placebo | - | 12 | 5 | ALL: 18-24 | ALL:  < 27 | - | No | Healthy | MPOD, Serum lutein and zeaxanthin |
|  |  | 7.44mg total macular carotenoid | L: 6.18 mg  Z: 0.73 mg  MZ: 0.53 mg | 12 | 7 | ALL: 18-25 |  | - |  |  |  |
|  |  | 13.13mg total macular carotenoid | L: 10.86 mg  Z: 1.33 mg  MZ: 0.94 mg | 12 | 8 | ALL: 18-26 |  | - |  |  |  |
|  |  | 27.03mg total macular carotenoid | L: 22.33 mg  Z: 2.70 mg  MZ: 2 mg | 12 | 8 | ALL: 18-27 |  | - |  |  |  |
| 47 | Stringham, NT (2019) | Placebo | - | 10 | 10 | ALL: 21.5 | ALL: 18.5-27 | ALL: 45.76 | No | Healthy | MPOD, Serum lutein and zeaxanthin |
|  |  | 13mg total MXans (Macular xanthophyll) | Total MXans: 13 mg | 24 | 24 | ALL: 21.5 |  |  |  |  |  |
|  |  | 27mg total total MXans (Macular xanthophyll) | Total MXans: 27 mg | 25 | 25 | ALL: 21.5 |  |  |  |  |  |
| 48 | Stringham,JM-EV (2016) | Placebo | - | 52 | 10 | ALL: 21.5 | ALL: <27 | ALL: 54.24 | No | Healthy | MPOD, CS, Serum lutein and zeaxanthin |
|  |  | 12 mg | Total carotenoids: 24mg L: 22.33mg Z: 2.70mg MZ: 2mg | 52 | 24 | ALL: 21.5 |  |  |  |  |  |
|  |  | 24mg | total carotenoids: 12mg L: 10.86mg Z: 1.33mg MZ: 0.94mg | 52 | 25 | ALL: 21.5 |  |  |  |  |  |
| 49 | Stringham,JM-Foods (2017) | Placebo | - | 26 | 13 | ALL: 21.2 | ALL: <27 | ALL : 47.92 | No | Healthy | MPOD, CS, Photostress recovery time |
|  |  | L, Z and MZ | L: 19.92mg Z: 2.4mg MZ: 1.68mg | 26 | 35 | ALL: 21.2 |  |  |  |  |  |
| 50 | Stringham,JM-VPPO(2016) | Placebo | - | 52 | 10 | ALL: 21.5 | ALL: <27 | ALL: 54.24 | No | Healthy | MPOD, CS, Serum lutein and zeaxanthin |
|  |  | 12 mg | Total carotenoids: 24mg L: 22.33mg Z: 2.70mg MZ: 2mg | 52 | 24 | ALL: 21.5 |  |  |  |  |  |
|  |  | 24mg | Total carotenoids: 12mg L: 10.86mg Z: 1.33mg MZ: 0.94mg | 52 | 25 | ALL: 21.5 |  |  |  |  |  |
| 51 | Tanito, M (2012) | Lutein | L: 10 mg of lutein  Z: 0.08 mg | 11 | 11 | 39.6 | - | 23.4 | Not mentioned | Healthy | MPOD |
|  |  | Zeaxanthin | Z:10mg | 11 | 11 | 38.3 | - | 23.4 |  |  |  |
| 52 | Thurnham (2015) | Normal-1 | L: 20mg Z: 2 mg  MZ: 0.3 mg | 12 | 12 | 59 | 28 | 27.3 | Yes (Not significant among groups) | Healthy | MPOD, Serum lutein, zeaxanthin and meso-zeaxanthin |
|  |  | Normal-2 | L: 10 mg Z: 2 mg  MZ: 10 mg | 10 | 10 | 60 | 26 | 36.8 |  |  |  |
|  |  | Normal-3 | L: 3 mg Z: 2 mg  MZ: 17 mg | 9 | 9 | 65 | 28 | 47.8 |  |  |  |
|  |  | AMD-1 | L: 20 mg  Z: 2 mg MZ: 0.3 mg | 11 | 11 | 67 | - | - |  | AMD |  |
|  |  | AMD-2 | L: 10 mg Z: 2 mg  MZ: 10 mg | 11 | 11 | 66 | - | - |  |  |  |
|  |  | AMD-3 | L: 3 mg  Z: 2 mg MZ: 17 mg | 10 | 10 | 65 | - | - |  |  |  |
| 53 | Weigert, G (2011) | Placebo (1-3m) | - | 42 | 42 | ALL: 21.5 | - | ALL: 43.1 | - | AMD | MPOD |
|  |  | Lutein (1-3m) | L:20mg | 84 | 84 |  | - |  |  |  |  |
|  |  | Placebo (4-6m) | - | - | - |  | - |  |  |  |  |
|  |  | Lutein (4-6m) | L: 10mg | - | - |  | - |  |  |  |  |
| 54 | Wolf-Schnurrbusch (2015) | Lutein | L: 10mg Vit C: 60mg Vit E: 20mg Niacin B3: 10mg Copper: 0.25mg Zinc: 10mg Zeaxanthine: 1mg | 40 | 40 | 75.2 | 25 | 45.0 | Yes (Sig not mentioned) | AMD | MPOD, VA, CS |
|  |  | Lutein + omega | L: 10mg Vit C: 60mg Vit E: 20mg Niacin B3: 10mg Copper: 0.25mg Zinc: 10mg Zeaxanthine: 1mg Omega-3: 160mg | 39 | 39 | 72.5 | 25 | 39.0 |  |  |  |
| 55 | Y, Yao (2013) | Placebo | - | 52 | 60 | 36.9 | 22.9 | 81.7 | No | Healthy | MPOD, CS |
|  |  | Active | L: 20mg | 52 | 60 | 36.5 | 24.4 | 83.3 |  |  |  |
| 56 | Yoshizako, H (2016) | Free lutein | Free L: 10mg L: 10mg | 13 | 10 | 33.8 | - | 30.0 | Yes (Sig not mentioned) | Healthy | MPOD, VA, CS, Serum lutein |
|  |  | Lutein ester | Lesters: 200mg L: 10mg | 13 | 10 | 30.7 | - | 50.0 |  |  |  |
| 57 | Yoshizako, K (2023) | Placebo | - | 8 | 29 | 30 | 22.2 | 93.1 | - | Healthy | MPOD |
|  |  | Astaxanthin + Lutein + Zeaxanthin | Astaxanthin: 6g containing  L: 10 mg Z: 2mg | 8 | 28 | 31 | 23.0 | 89.2 |  |  |  |
| 58 | Yoshizako, T (2023) | Placebo | - | 96 | 13 | 47 | - | 7.7 | - | High myopia | MPOD, VA, CS |
|  |  | Lutein | L: 20mg | 96 | 15 | 43 | - | 20.0 |  |  |  |
| 59 | Zanón-Moreno (2021) | Lutein | Vit C: 80 mg Vit E: 12mg B1: 1.1 mg B2: 1.4 mg B3: 16mg B6: 1.4mg B9: 200ug B12: 2.5ug Zn: 7.5mg Cu: 1mg se: 55ug Mn: 2 mg L: 6mg ZX: 0.3mg Glutathione: 1mg | 13 | 49 | 44.6 | - | 51.1 | - | Healthy | MPOD, Plasma lutein |
|  |  | Lutein + DHA | DHA: 700mg EPA: 85mg DPA: 60mg VitC: 53.4mg Vit E: 8mg B1: 0.74mg B2: 0.94mg B3: 10.6mg B6: 0.94mg B9: 133.4ug B12: 1.66ug Zn: 3.32mg Cu: 0.32mg se: 18.32ug Mn: 0.66mg L: 6mg ZX: 0.6mg Glutathione: 4mg | 13 | 51 | 44 | - | 40.8 |  |  |  |
| 60 | Zhang, Y (2022) | Blank Control | - | 13 | 18 | - | - | - | - | High myopia | MPOD |
|  |  | Low-dose lutein control | L: 1mg | 13 | 9 | - | - | - |  |  |  |
|  |  | High-dose lutein control | L: 2mg | 13 | 9 | - | - | - |  |  |  |

^1^Mixure of Antixodiants: Vit C: 500mg; Thiamin: 1.5mg; Riboflavin: 1.7mg; Niacin: 20mg; Vit B6: 10mg; Folate: 800mcg; Pathothenic acid: 10mg; Magnesium: 100mg; Zinc: 25mg; Vit B12: 1000mcg; Vit D3: 2000 IU; Vit E: 200 IU; Biotin: 100mcg; Selenium: 70mg; Copper: 3mg; Manganese: 2mg; Chromium: 120 mcg; Molybdenum: 75mcg; NAC: 500mg; POA Blend: 200mg; Acetyl-L-Carnitine: 500mg; Taurine: 500mg; Quercetin: 100mg; CoQ10: 50mg; Lycopene: 500mcg

**Supplemental Table 3.** Details for Network Diagram (Macular Pigment Optical Density)

| **Treatment** | **Number of Direct Comparisons** | **Number of Subjects** |
| --- | --- | --- |
| L+Z vs. L+Z+MZ | 10 | 249 |
| L vs. Placebo | 8 | 350 |
| L+Z vs. Placebo | 6 | 278 |
| MIX+L+Z vs. Placebo | 7 | 728 |
| L+Z+MZ vs. Placebo | 5 | 198 |
| L vs. L+Z | 4 | 207 |
| MIX+FA+L+Z vs. Placebo | 3 | 331 |
| L+Z+FA vs. Placebo | 2 | 84 |
| L vs. Z | 1 | 19 |
| L+Z vs. L+Z+FA | 1 | 79 |
| L+Z+FA vs. Z | 1 | 54 |
| MIX+FA+L+Z vs. MIX+L+Z | 1 | 41 |
| Placebo vs. Z | 1 | 44 |

**Supplemental Table 4.** Details for Network Diagram (Visual Acuity)

| **Treatment** | **Number of Direct Comparisons** | **Number of Subjects** |
| --- | --- | --- |
| L vs. Placebo | 8 | 496 |
| L vs. L+Z | 4 | 214 |
| L+Z vs. Placebo | 4 | 218 |
| MIX+L+Z vs. Placebo | 3 | 216 |
| L+Z+MZ vs. Placebo | 2 | 140 |
| MIX+FA+L+Z vs. Placebo | 2 | 92 |
| FA+L+Z vs. Placebo | 1 | 44 |
| L vs. MIX+FA+L | 1 | 59 |
| MIX+FA+L vs. Placebo | 1 | 61 |
| MIX+FA+L+Z vs. MIX+L+Z | 1 | 41 |

**Supplemental Table 5.** Details for Network Diagram (Contrast Sensitivity-Low Spatial Frequency)

| **Treatment** | **Number of Direct Comparisons** | **Number of Subjects** |
| --- | --- | --- |
| L vs. Placebo | 5 | 244 |
| L vs. L+Z | 4 | 214 |
| L+Z vs. L+Z+MZ | 4 | 121 |
| L+Z vs. Placebo | 4 | 218 |
| MIX+FA+L+Z vs. Placebo | 2 | 92 |
| FA+L+Z vs. Placebo | 1 | 44 |
| L+Z+FA vs. Placebo | 1 | 40 |
| L+Z+FA vs. Z | 1 | 54 |
| L+Z+MZ vs. Placebo | 1 | 78 |
| MIX+FA+L+Z vs. MIX+L+Z | 1 | 41 |
| MIX+L+Z vs. Placebo | 1 | 31 |
| Placebo vs. Z | 1 | 44 |

**Supplemental Table 6.** Details for Network Diagram (Contrast Sensitivity-Normal Spatial Frequency)

| **Treatment** | **Number of Direct Comparisons** | **Number of Subjects** |
| --- | --- | --- |
| L vs. Placebo | 5 | 242 |
| L vs. L+Z | 4 | 214 |
| L+Z vs. L+Z+MZ | 4 | 121 |
| L+Z vs. Placebo | 4 | 218 |
| L+Z+FA vs. Placebo | 1 | 40 |
| L+Z+FA vs. Z | 1 | 54 |
| L+Z+MZ vs. Placebo | 1 | 78 |
| MIX+FA+L+Z vs. MIX+L+Z | 1 | 41 |
| MIX+FA+L+Z vs. Placebo | 1 | 40 |
| MIX+L+Z vs. Placebo | 1 | 31 |
| Placebo vs. Z | 1 | 44 |

**Supplemental Table 7.** Details for Network Diagram (Contrast Sensitivity-High Spatial Frequency)

| **Treatment** | **Number of Direct Comparisons** | **Number of Subjects** |
| --- | --- | --- |
| L vs. Placebo | 5 | 242 |
| L vs. L+Z | 4 | 214 |
| L+Z vs. L+Z+MZ | 4 | 121 |
| L+Z vs. Placebo | 4 | 216 |
| L+Z+FA vs. Placebo | 1 | 40 |
| L+Z+FA vs. Z | 1 | 54 |
| L+Z+MZ vs. Placebo | 1 | 78 |
| MIX+FA+L+Z vs. MIX+L+Z | 1 | 39 |
| MIX+FA+L+Z vs. Placebo | 1 | 38 |
| MIX+L+Z vs. Placebo | 1 | 31 |
| Placebo vs. Z | 1 | 44 |

**Supplemental Table 8.** Details for Network Diagram (Photostress Recovery Time)

| **Treatment** | **Number of Direct Comparisons** | **Number of Subjects** |
| --- | --- | --- |
| L vs. L+Z | 4 | 214 |
| L vs. Placebo | 4 | 216 |
| L+Z vs. Placebo | 4 | 218 |
| L+Z+MZ vs. Placebo | 1 | 48 |

| Placebo |  |  |  |
| --- | --- | --- | --- |
| 5.56 (1.69 to 8.80) | **L** |  |  |
| 5.73 (1.46 to 8.76) | 0.16 (-3.45 to 3.33) | **L+Z** |  |
| 3.19 (-2.17 to 8.61) | -2.37 (-8.28 to 4.63) | -2.54 (-8.25 to 4.87) | **L+Z+MZ** |

**Supplemental Table 9.** League Table (Photostress Recovery Time)

**Supplemental Table 10.** League Table (Contrast Sensitivity-Low Spatial Frequency)

| Placebo |  |  |  |  |  |  |  |
| --- | --- | --- | --- | --- | --- | --- | --- |
| -0.14 (-0.23 to -0.05) | **L** |  |  |  |  |  |  |
| -0.14 (-0.22 to -0.05) | 0.01 (-0.09 to 0.10) | **L+Z** |  |  |  |  |  |
| -0.16 (-0.40 to -0.00) | -0.01 (-0.28 to 0.16) | -0.02 (-0.28 to 0.15) | **L+Z+FA** |  |  |  |  |
| -0.18 (-0.29 to -0.08) | -0.04 (-0.16 to 0.08) | -0.05 (-0.13 to 0.04) | -0.03 (-0.20 to 0.24) | **L+Z+MZ** |  |  |  |
| -0.00 (-0.13 to 0.13) | 0.14 (-0.02 to 0.30) | 0.14 (-0.02 to 0.29) | 0.15 (-0.03 to 0.44) | 0.18 (0.02 to 0.35) | **MIX+FA+L+Z** |  |  |
| -0.01 (-0.18 to 0.16) | 0.13 (-0.06 to 0.32) | 0.12 (-0.06 to 0.32) | 0.14 (-0.07 to 0.45) | 0.17 (-0.03 to 0.38) | -0.01 (-0.18 to 0.16) | **MIX+L+Z** |  |
| -0.13 (-0.35 to 0.03) | 0.01 (-0.22 to 0.19) | 0.00 (-0.22 to 0.18) | 0.02 (-0.14 to 0.23) | 0.05 (-0.19 to 0.24) | -0.13 (-0.38 to 0.07) | -0.12 (-0.40 to 0.11) | **Z** |

**Supplemental Table 11.** League Table (Contrast Sensitivity-Normal Spatial Frequency)

| Placebo |  |  |  |  |  |  |  |
| --- | --- | --- | --- | --- | --- | --- | --- |
| -0.21 (-0.32 to -0.11) | **L** |  |  |  |  |  |  |
| -0.16 (-0.26 to -0.06) | 0.06 (-0.05 to 0.16) | **L+Z** |  |  |  |  |  |
| -0.03 (-0.24 to 0.18) | 0.19 (-0.05 to 0.42) | 0.13 (-0.10 to 0.36) | **L+Z+FA** |  |  |  |  |
| -0.18 (-0.31 to -0.06) | 0.04 (-0.11 to 0.18) | -0.02 (-0.12 to 0.08) | -0.15 (-0.39 to 0.09) | **L+Z+MZ** |  |  |  |
| -0.09 (-0.30 to 0.12) | 0.13 (-0.11 to 0.36) | 0.07 (-0.16 to 0.30) | -0.06 (-0.36 to 0.24) | 0.09 (-0.16 to 0.34) | **MIX+FA+L+Z** |  |  |
| -0.02 (-0.24 to 0.19) | 0.19 (-0.05 to 0.42) | 0.13 (-0.10 to 0.37) | 0.01 (-0.30 to 0.31) | 0.15 (-0.09 to 0.41) | 0.07 (-0.15 to 0.28) | **MIX+L+Z** |  |
| -0.14 (-0.35 to 0.06) | 0.08 (-0.16 to 0.31) | 0.02 (-0.21 to 0.25) | -0.11 (-0.32 to 0.10) | 0.04 (-0.20 to 0.28) | -0.05 (-0.34 to 0.24) | -0.11 (-0.42 to 0.18) | **Z** |

**Supplemental Table 12.** League Table (Contrast Sensitivity-High Spatial Frequency)

| Placebo |  |  |  |  |  |  |  |
| --- | --- | --- | --- | --- | --- | --- | --- |
| -0.04 (-0.13 to 0.06) | **L** |  |  |  |  |  |  |
| -0.03 (-0.12 to 0.06) | 0.01 (-0.09 to 0.10) | **L+Z** |  |  |  |  |  |
| -0.07 (-0.25 to 0.12) | -0.03 (-0.24 to 0.17) | -0.04 (-0.24 to 0.17) | **L+Z+FA** |  |  |  |  |
| -0.05 (-0.16 to 0.06) | -0.02 (-0.14 to 0.10) | -0.02 (-0.11 to 0.07) | 0.02 (-0.20 to 0.23) | **L+Z+MZ** |  |  |  |
| -0.10 (-0.29 to 0.09) | -0.07 (-0.28 to 0.14) | -0.07 (-0.28 to 0.13) | -0.03 (-0.30 to 0.23) | -0.05 (-0.27 to 0.17) | **MIX+FA+L+Z** |  |  |
| -0.07 (-0.26 to 0.13) | -0.03 (-0.25 to 0.18) | -0.04 (-0.25 to 0.17) | 0.00 (-0.26 to 0.27) | -0.01 (-0.24 to 0.21) | 0.03 (-0.16 to 0.23) | **MIX+L+Z** |  |
| -0.07 (-0.25 to 0.12) | -0.03 (-0.24 to 0.17) | -0.04 (-0.24 to 0.17) | -0.00 (-0.19 to 0.19) | -0.02 (-0.23 to 0.20) | 0.03 (-0.23 to 0.30) | 0.00 (-0.27 to 0.27) | **Z** |

**Supplemental Table 13.** Quality of Evidence based on GRADE (Grading of Recommendations, Assessment, Development and Evaluations) evidence assessment.^*^

| **Outcome (No. of studies)** | **Quality Assessment** | | | | | **Overall Quality of Evidence** |
| --- | --- | --- | --- | --- | --- | --- |
|  | **Limitations of study design** | **Inconsitency** | **Indirectness** | **Imprescision** | **Publication bias** |  |
| **Macular Pigment Optical Density (29)** | Serious (-1) | Not Serious | Serious (-1) | Not Serious | Undetected | ⊕⊕◯◯  Low |
| **Visual Acuity (12)** | Not Serious | Not Serious | Serious (-1) | Not Serious | Detected (-1) | ⊕⊕◯◯  Low |
| **Contrast Sensitivity-Low Spatial Frequency (10)** | Not Serious | Not Serious | Serious (-1) | Not Serious | Detected (-1) | ⊕⊕◯◯  Low |
| **Contrast Sensitivity-Normal Spatial Frequency (8)** | Not Serious | Not Serious | Serious (-1) | Not Serious | Undetected | ⊕⊕⊕◯  Moderate |
| **Contrast Sensitivity-High Spatial Frequency (8)** | Not Serious | Not Serious | Serious (-1) | Not Serious | Detected (-1) | ⊕⊕◯◯  Low |
| **Photostress Recovery Time (4)** | Not Serious | Serious (-1) | Serious (-1) | Not Serious | Undetected | ⊕⊕◯◯  Low |

^*^ "(-1)" signifies a one-level downgrading of evidence quality in the GRADE approach.

**Supplemental Figure 1.** Heterogeneity Assessment for Macular Pigment Optical Density with respect to potential effect modifiers: A)age; B) body mass index; C) study duration (weeks); D) gender (% male); F) sample size. Error bar indicates mean ± SD when applicable.

**Supplemental Figure 2.** Heterogeneity Assessment for Visual Acuity with respect to potential effect modifiers: A) age; B) body mass index; C) study duration (weeks); D) gender (% male); F) sample size. Error bar indicates mean ± SD when applicable.


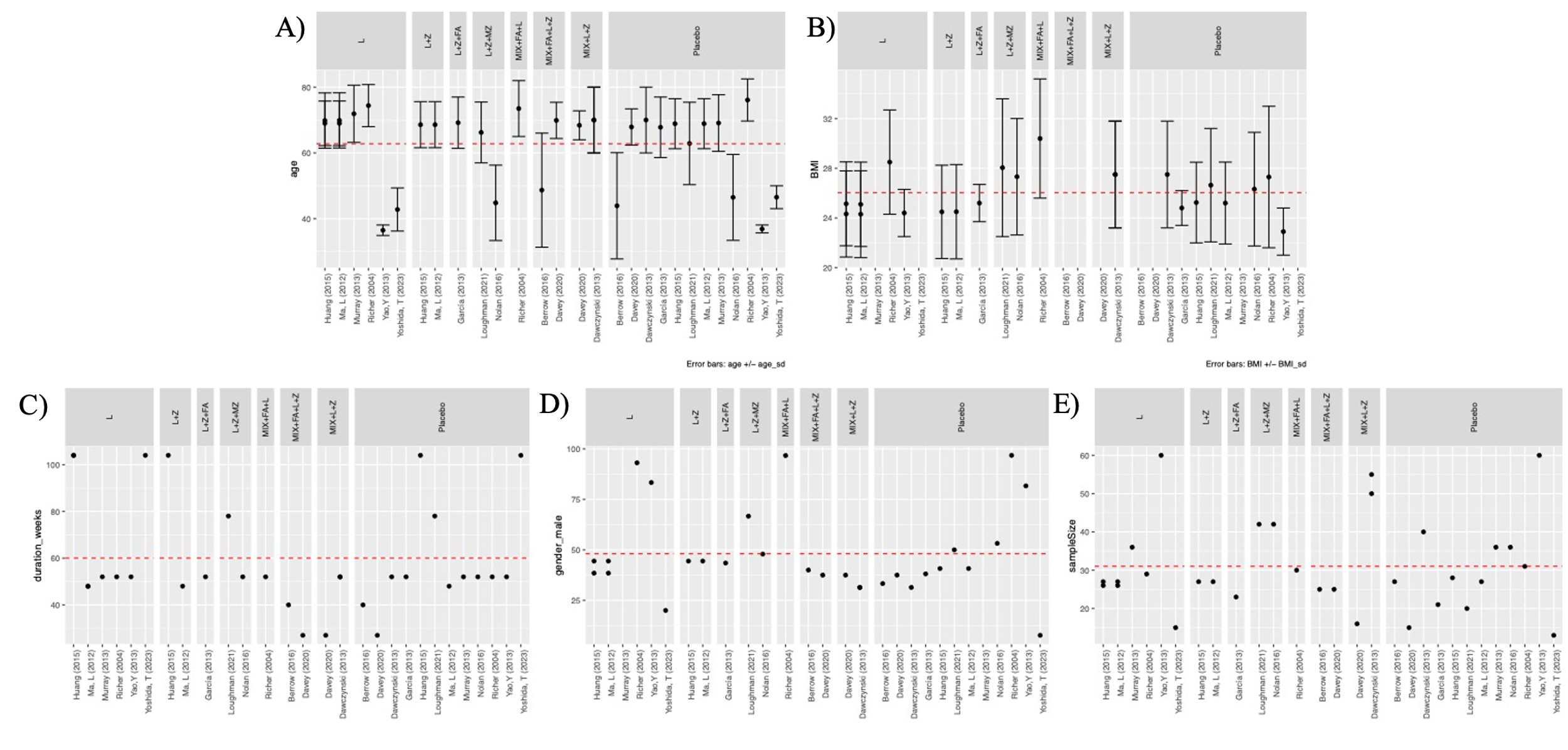


**Supplemental Figure 3.** Heterogeneity Assessment for Contrast Sensitivity-Low Spatial Frequency with respect to potential effect modifiers: A) age; B) body mass index; C) study duration (weeks); D) gender (% male); F) sample size. Error bar indicates mean ± SD when applicable.


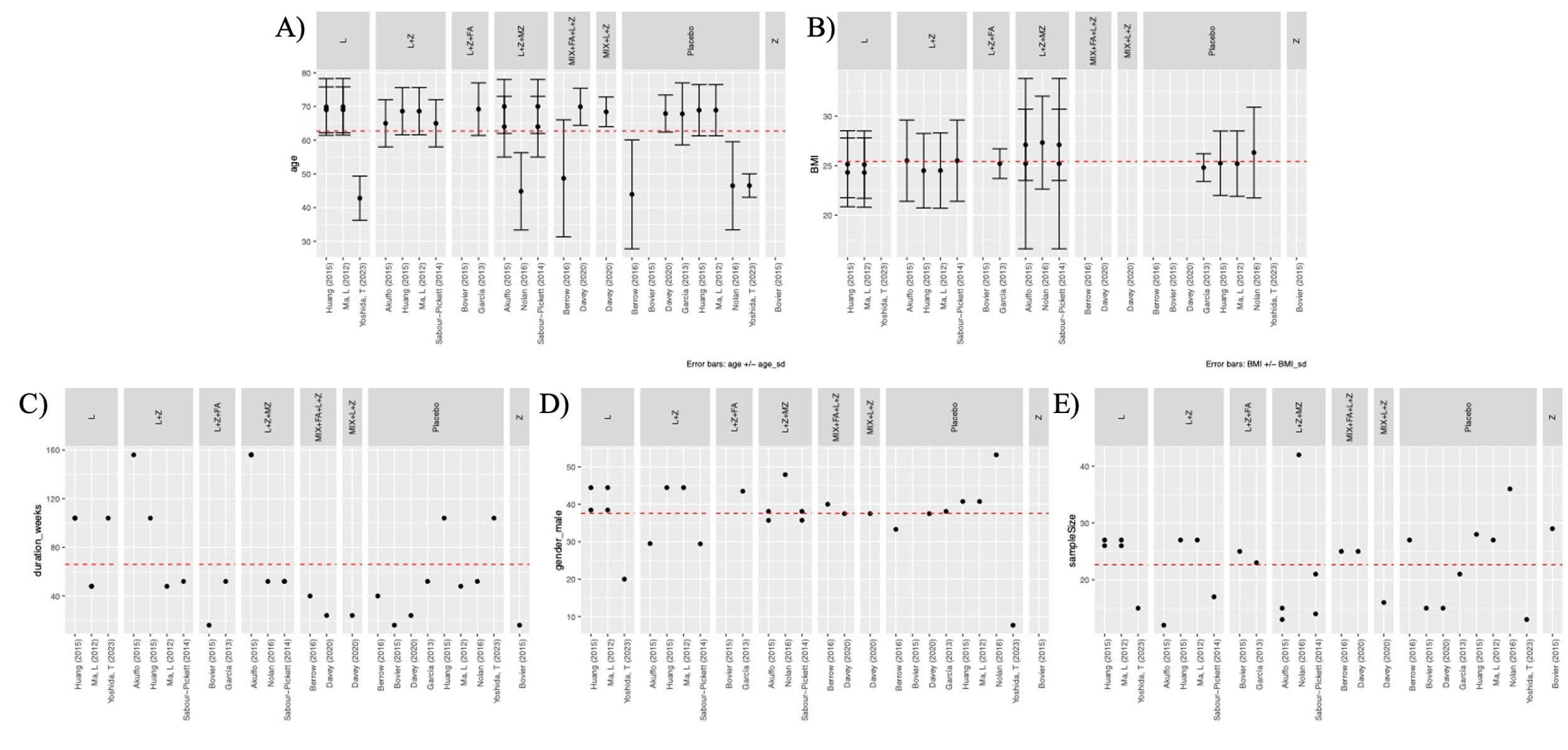


**Supplemental Figure 4.** Heterogeneity Assessment for Contrast Sensitivity-Normal Spatial Frequency with respect to potential effect modifiers: A) age; B) body mass index; C) study duration (weeks); D) gender (% male); F) sample size. Error bar indicates mean ± SD when applicable.


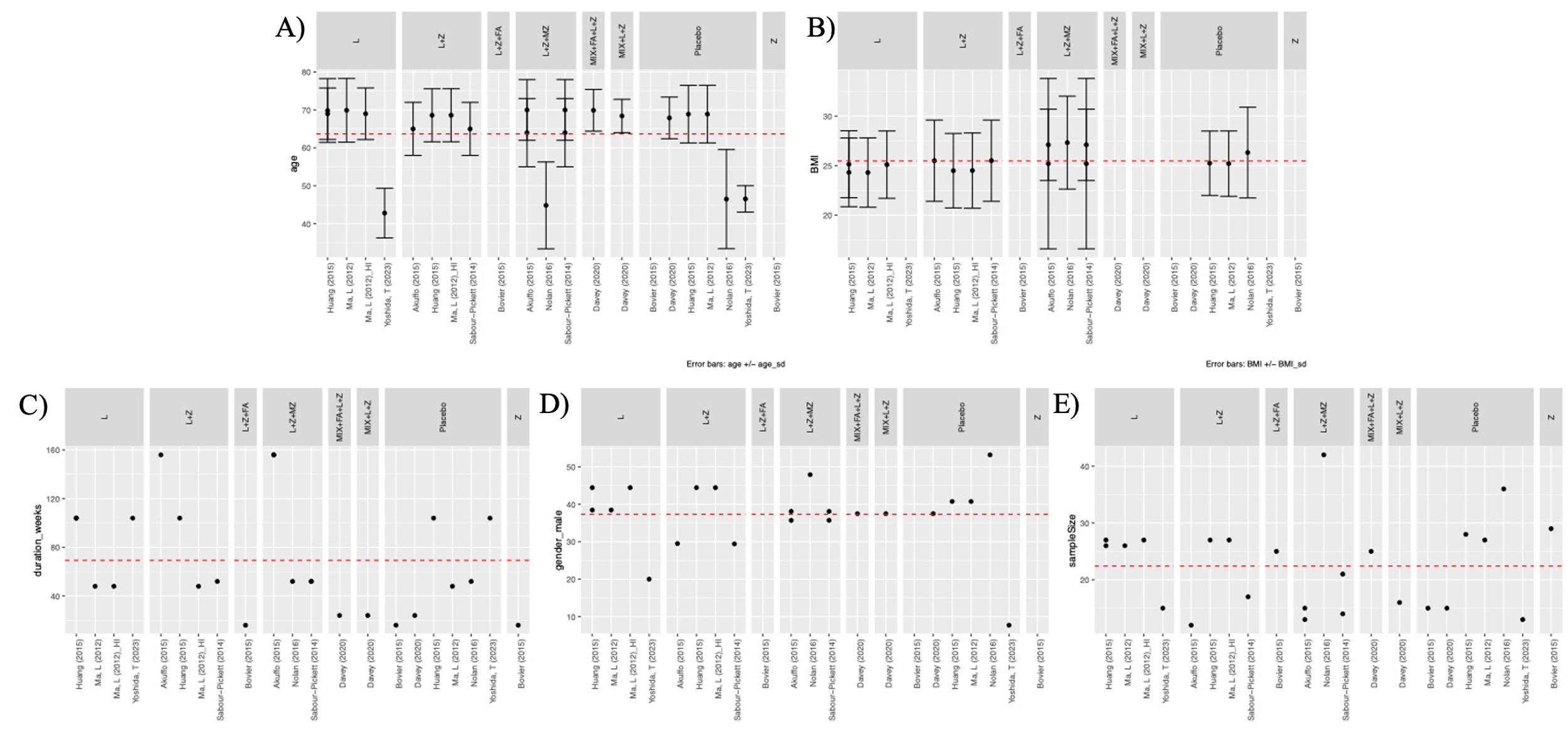


**Supplemental Figure 5.** Heterogeneity Assessment for Contrast Sensitivity-High Spatial Frequency with respect to potential effect modifiers: A) age; B) body mass index; C) study duration (weeks); D) gender (% male); F) sample size. Error bar indicates mean ± SD when applicable.


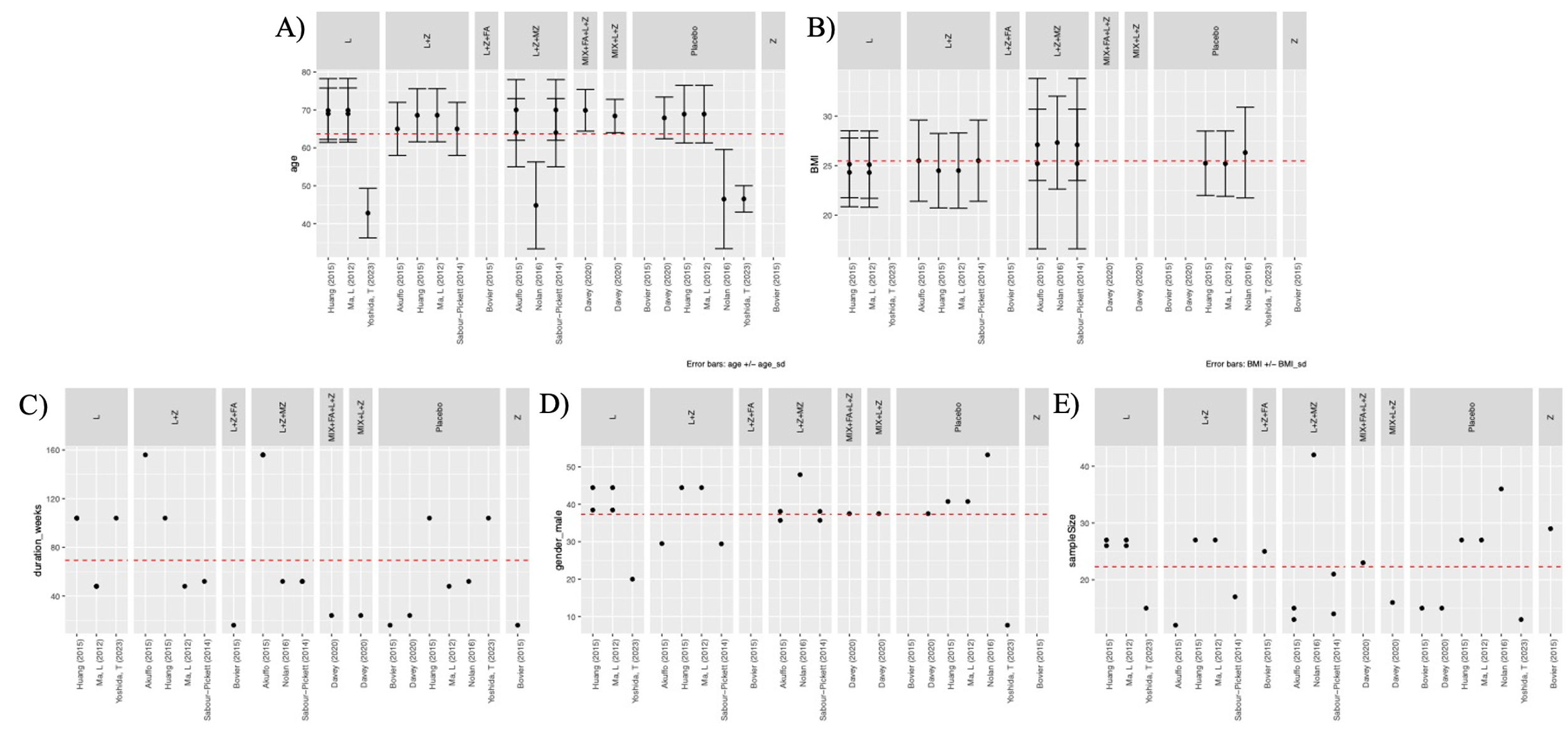


**Supplemental Figure 6.** Heterogeneity Assessment for Photostree Recovery Time with respect to potential effect modifiers: A) age; B) body mass index; C) study duration (weeks); D) gender (% male); F) sample size. Error bar indicates mean ± SD when applicable.


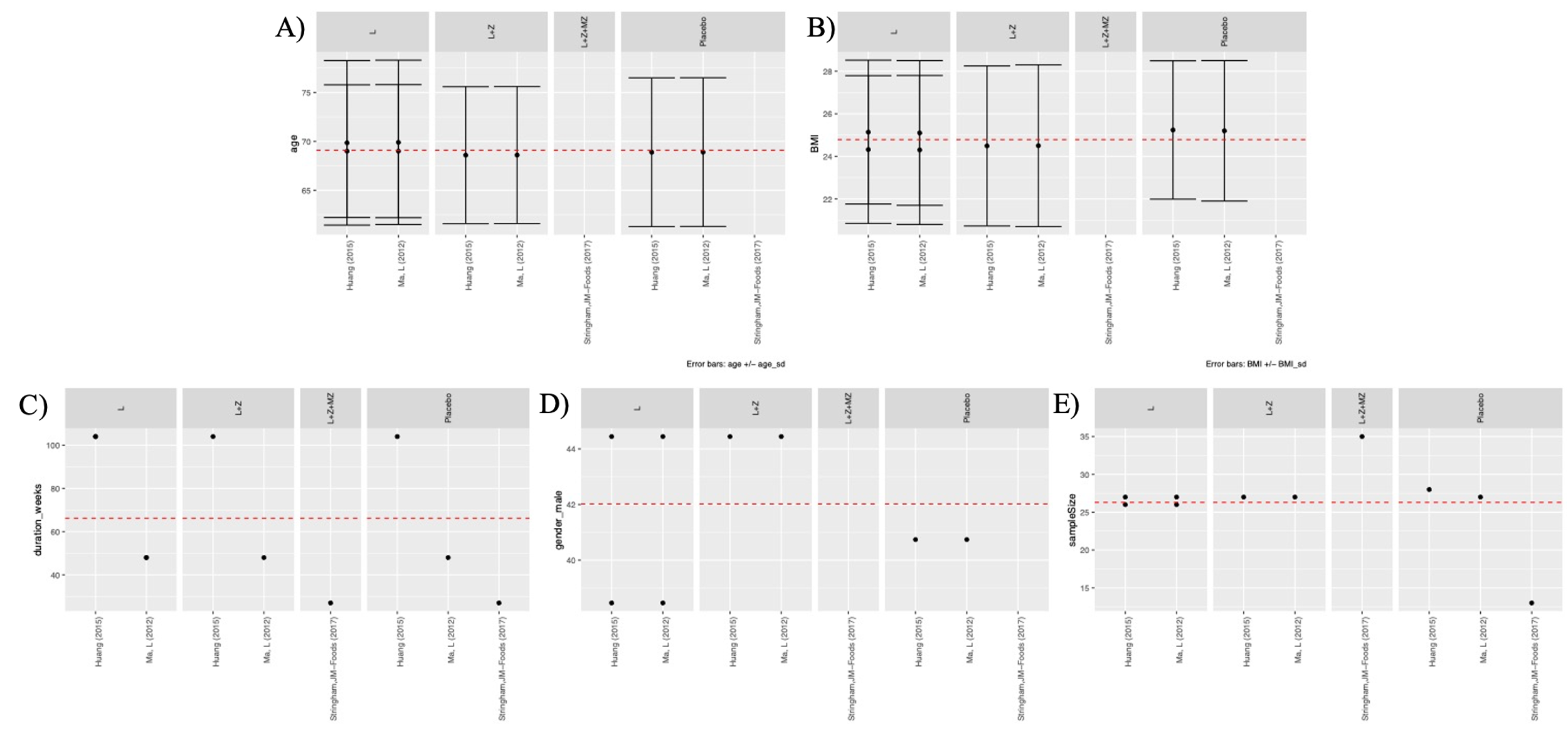


**Supplemental Figure 7.** Inconsistency and Consistency Model Fitting (Macular Pigment Optical Density)

**Supplemental Figure 8.** Inconsistency and Consistency Model Fitting (Visual Acuity)

**Supplemental Figure 9.** Inconsistency and Consistency Model Fitting (Contrast Sensitivity-Low Spatial Frequency)

**Supplemental Figure 10.** Inconsistency and Consistency Model Fitting (Contrast Sensitivity-Normal Spatial Frequency)

**Supplemental Figure 11.** Inconsistency and Consistency Model Fitting (Contrast Sensitivity-High Spatial Frequency)

**Supplemental Figure 12.** Inconsistency and Consistency Model Fitting (Photostress Recovery Time)

**Supplemental Figure 13.** Comparison-adjusted Funnel Plots (Macular Pigment Optical Density)

**Supplemental Figure 14.** Comparison-adjusted Funnel Plots (Visual Acuity)

**Supplemental Figure 15.** Comparison-adjusted Funnel Plots (Contrast Sensitivity-Low Spatial Frequency)

**Supplemental Figure 16.** Comparison-adjusted Funnel Plots (Contrast Sensitivity-Normal Spatial Frequency)

**Supplemental Figure 17.** Comparison-adjusted Funnel Plots (Contrast Sensitivity-High Spatial Frequency)

**Supplemental Figure 18.** Comparison-adjusted Funnel Plots (Photostress Recovery Time)

**Supplemental Figure 19.** Summary of Risk of Bias Assessment
